# Pulse-doubling perovskite nanowire lasers enabled by phonon-assisted multistep energy funneling

**DOI:** 10.1038/s41377-024-01494-2

**Published:** 2024-07-17

**Authors:** Chunhu Zhao, Jia Guo, Jiahua Tao, Junhao Chu, Shaoqiang Chen, Guichuan Xing

**Affiliations:** 1 Hunan Provincial Key Laboratory of Carbon Neutrality and Intelligent Energy, School of Resource & Environment, Hunan University of Technology and Business, 410205 Changsha, China; 2 Engineering Research Center for Nanophotonics and Advanced Instrument, Ministry of Education, School of Physics and Electronic Science, East China Normal University, 200241 Shanghai, China; 3 Joint Key Laboratory of the Ministry of Education, Institute of Applied Physics and Materials Engineering, University of Macau, 999078 Macau, China

**Keywords:** Micro-optics, Nonlinear optics

## Abstract

Laser pulse multiplication from an optical gain medium has shown great potential in miniaturizing integrated optoelectronic devices. Perovskite multiple quantum wells (MQWs) structures have recently been recognized as an effective gain media capable of doubling laser pulses that do not rely on external optical equipment. Although the light amplifications enabled with pulse doubling are reported based on the perovskite MQWs thin films, the micro-nanolasers possessed a specific cavity for laser pulse multiplication and their corresponding intrinsic laser dynamics are still inadequate. Herein, a single-mode double-pulsed nanolaser from self-assembled perovskite MQWs nanowires is realized, exhibiting a pulse duration of 28 ps and pulse interval of 22 ps based on single femtosecond laser pulse excitation. It is established that the continuous energy building up within a certain timescale is essential for the multiple population inversion in the gain medium, which arises from the slowing carrier localization process owning to the stronger exciton–phonon coupling in the smaller-*n* QWs. Therefore, the double-pulsed lasing is achieved from one fast energy funnel process from the adjacent small-*n* QWs to gain active region and another slow process from the spatially separated ones. This report may shed new light on the intrinsic energy relaxation mechanism and boost the further development of perovskite multiple-pulse lasers.

## Abstract (web summary)

Perovskite multiple quantum wells are promising gain mediums for laser pulse multiplication. Here, the authors demonstrate a perovskite double-pulsed nanowire laser with pulse interval of 22 ps.

## Graphical abstract

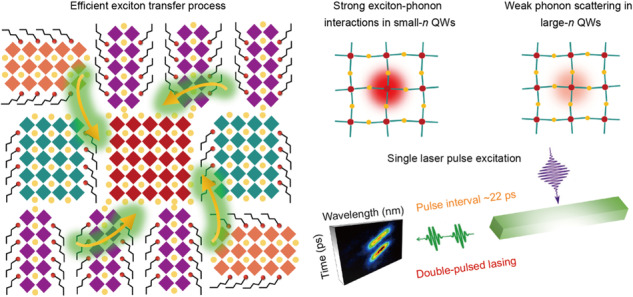

## Introduction

Laser pulse multiplication is a process whereby the single laser pulse generates double or multiple laser pulses via specific methods, such as pulse stacker, pulse shaper, pulse splitter, optical delay line and interferometer^1–7^. However, these methods typically rely on external optical modules that increase the bulkiness, complexity and cost of multiple-pulse laser sources, limiting future miniaturization and integration. Fortunately, recent advances in ultrashort laser pulse multiplication by using perovskite MQWs^8^ open up new possibilities for the fabrication of nanoscale laser pulse doubling devices. Unlike the external optical modulation category, perovskite MQWs as gain medium present unique two-step energy funnels for double-pulsed stimulated emission (StE). Indeed, such unique optical properties, as well as large exciton binding energy and better stability^9,10^ make perovskite MQWs more attractive gain materials for laser applications than 3D perovskites. In the past few years, extensive efforts have been made to explore versatile perovskite MQWs lasers^11–14^; however, most are concentrated on the demonstration of lasing properties based on compositional engineering, and there has been no further progress towards double-pulsed nanowire lasers.

Typically, perovskite MQWs with the general formula of L_2_A_*n*−1_M_*n*_X_3*n*+1_ are composed of the corner-sharing inorganic [MX_6_] octahedral layers with different thicknesses (*n*), which are sandwiched by two long-chain organic spacers (L). Under laser pulse excitation, stable excitons with large binding energies are primarily generated in small-*n* QWs (*n* ≤ 3)^15,16^ due to strong quantum and dielectric confinement^17,18^ and then localized into large-*n* QWs (*n* = ∞). The ultrafast exciton energy transfer processes allow efficient radiative recombination and avoid the exciton quenching effect, benefiting the build-up of population inversion for low threshold lasers^19–25^. The energy domain distribution and lattice vibrations through electron–phonon coupling can strongly affect the energy transfer process. In the perovskite MQWs, the randomly stacked small-*n* QWs that are prone to form due to their low formation energy^26^ produce inhomogeneous energy domain distribution, thus leading to a multi-channel energy transfer process^27^. This is mainly because the QW width determines the magnitude of excitonic binding energy and carrier dynamics^28,29^. Although inhomogeneous energy domain distribution is detrimental to the efficiency of photovoltaics and light-emitting diodes^30–33^, it may provide essential laser gain conditions for modifying or multiplying the output laser pulses. For instance, the observation of dual-pulsed lasing from the perovskite MQW thin films was reported, benefiting from multistep energy transfer with one fast vertical and another slow lateral transfer process^8^. Electron–phonon coupling plays a critical role in the light emission of perovskites, expected to either provide polaronic protection of charge carriers or broaden emission^34,35^. From the point of energy transfer, phonon scattering sets a basic intrinsic limit to carrier mobility^36,37^, thereby affecting carrier recombination dynamics in the perovskites. However, the view on the relationship between phonon scattering and the multistep energy transfer process towards the laser pulse multiplication has been lacking until now.

In this work, we demonstrate the double-pulsed lasing from (BA)_2_Cs_*n*−1_Pb_*n*_Br_3*n*+1_ quasi-2D perovskite nanowires under single 400 nm femtosecond laser pulse excitation. The nanowire lasers possess a low threshold of 7.87 μJ cm^−2^ and double laser pulses with a pulse interval of around 22 ps, which arise from one fast energy funnel channel from the adjacent small-*n* QWs to gain active region and another slow process from the spatially separated ones. We then investigate the critical role of exciton–phonon interactions on the multistep carrier funneling process, as evidenced by temperature-dependent photoluminescence (PL) and time-resolved PL (TRPL) measurements. Strong exciton–phonon coupling in small-*n* QWs prolongs the timescale of the carrier injection into gain medium, therefore facilitating the generation of doubling laser pulses. These insights pave the way towards the development of low threshold ultrashort double-pulsed on-chip optoelectronic devices.

## Results

### Quasi-2D perovskite nanowires

**Fig. 1:**
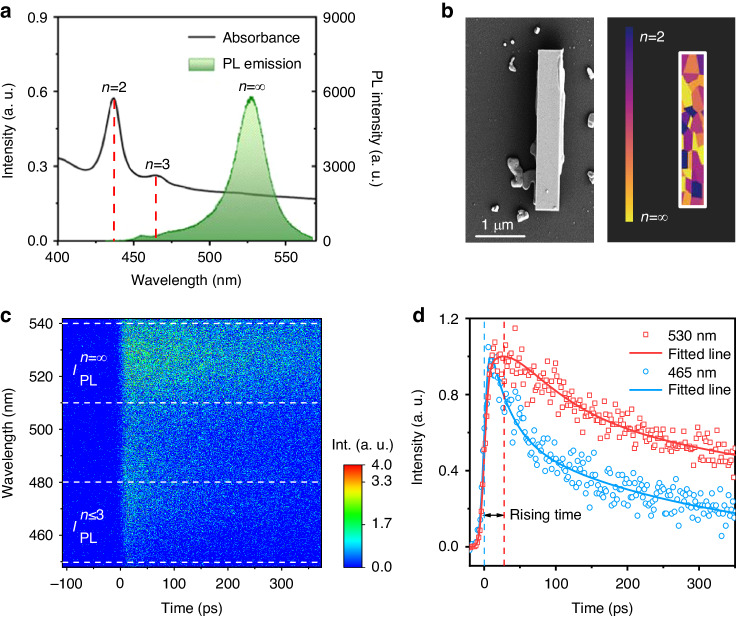
Structure characterization of (BA)_2_Cs_*n*−1_Pb_*n*_Br_3*n*+1_ perovskite nanowires. **a** The absorption spectrum (black line) of numerous 2D nanowires measured by UV–vis spectroscopy, and the PL spectrum (green line) under 400 nm femtosecond laser excitation. **b** SEM image and schematic diagram of as-grown nanowire with MQWs structure. The scale bar is 1 µm. **c** Pseudo-color TRPL plots at low pump fluence show spectra evolution along the decay time of 2D nanowires. **d** The normalized PL decay curves extracted at the small-*n* QWs and large-*n* QWs emission position, which clearly shows the building up exciton resonance at the large-*n* QWs (with excitation at 400 nm, 1 kHz, 50 fs, with pump fluence of 1.80 μJ cm^−2^)

In our experiments, (BA)_2_Cs_*n*−1_Pb_*n*_Br_3*n*+1_ perovskite nanowires are synthesized on the substrates by using a precursor solution self-assembled crystallization method at the ambient air (see “Materials and methods” section for details). These perovskite nanowires consist of multiple thickness QW structures with the dominated component of *n* = 2, as determined by ultraviolet and visible (UV–vis) absorption spectrum (Fig. 1a). For morphological measurement and componential analysis, scanning electron microscopy (SEM) and corresponding energy-dispersive X-ray spectroscopy (EDS) mapping of a single nanowire is performed (Figs. 1b and S1). The nanowire typically has a width of about 0.6 μm and a length of about 4.3 μm, showing well-defined wire-like morphology with smooth surfaces and flat end facets, which can serve as Fabry–Pérot (FP) cavity to offer sufficient coherent optical feedback for laser oscillations.

To examine their PL behaviors, the nanowires are excited by a 400 nm femtosecond laser pulse. The result clearly shows that the PL from 2D nanowire is dominated by the recombination in large-*n* QWs (*n* = ∞) at low injected carrier density. As the pump fluence increases, the electronic states in large-*n* QWs will be partially saturated, which impedes the further exciton localization from small-*n* QWs to large-*n* QWs (see Fig. S2). Hence, the high-energy PL peaks from the small-*n* QWs gradually emerge. We further demonstrate the exciton localization process with TRPL measurements at low carrier excitation densities. As shown in Fig. 1c, d, the TRPL curves extracted from the emission position of small-*n* QWs (*n* ≤ 3) and large-*n* QWs (*n* = ∞) clearly show that the fast decay of exciton PL at the small-*n* QWs, which is consistent with the corresponding rise of the PL signal at the large-*n* QWs’ resonances. This indicates that a substantial portion of the photo-generated exciton is localized from the small-*n* QWs to the large-*n* QWs, and the lifetime of this localization process is ~25 ± 5 ps when the injected charge carrier density is around 10^17^ cm^-3^ (Figs. S3 and S4). Such carrier density-dependent spectra evolution provides clear evidence for the high-efficiency exciton transfer from the small-*n* QWs to the large-*n* QWs, and the schematic of the energy transfer process is presented in Fig. S5.

### Lasing emission from quasi-2D perovskite nanowires

**Fig. 2:**
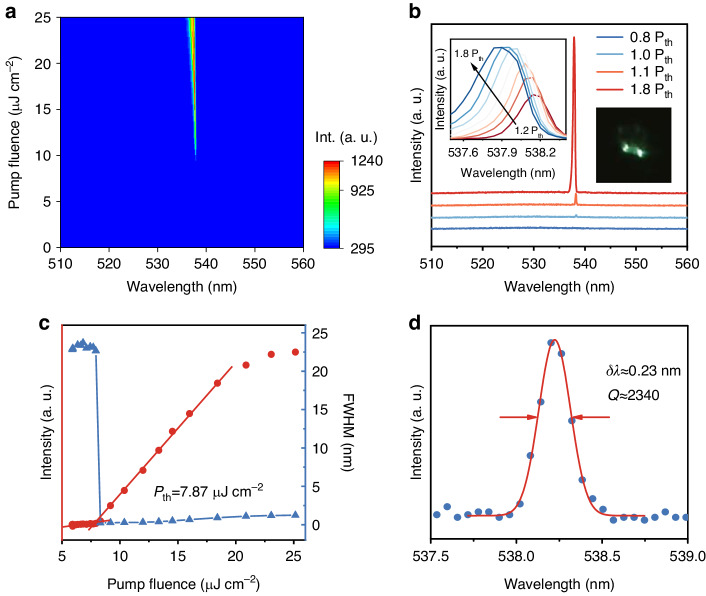
Single mode lasing from quasi-2D perovskite nanowires. **a** 2D pseudo-color plot of emission spectra with different pump fluences. **b** Evolution of lasing spectra before and after the lasing threshold. Inset: The blueshift of emission wavelength at the higher pump fluence. **c** Pump fluence-dependent PL intensities and FWHM of the emission bands. **d** Gaussian fitting of the individual lasing peak near the lasing threshold

In order to investigate the optical microcavity effect, the optically pumped lasing experiments of as-grown quasi-2D perovskite nanowires are performed with the confocal µ-PL system by a Ti:sapphire femtosecond pulse laser. The pulsed laser beam (400 nm, 50 fs, 1 kHz) with the beam waist is adjusted to be larger than the length of each nanowire. Figure 2a shows a 2D pseudo-color plot of the emission spectra versus pump fluences. Representative PL spectra below and above the lasing threshold are shown in Fig. 2b. At low pump fluence of 5.88 μJ cm^−2^, spontaneous emission characteristics centered at 529 nm are observed with full width at half maximum (FWHM) of 22 nm. Accompanied by the increased pump fluences, two bright emissive edges (see the inset of Fig. 2b) and sharp emission peaks occur to show spatial interference patterns, further demonstrating the formation of coherent lasing emission.

When the pump fluence reached the corresponding lasing threshold (*P*_th_ = 7.87 μJ cm^−2^, shown in Fig. 2c), strong single-mode lasing emission emerges at the red side of the PL peak around 539 nm, and the FWHM of the lasing peak decreases dramatically to 0.23 nm. With increasing pump fluence above the lasing threshold, the blueshift of the lasing peak ranging from 538.2 to 537.9 nm occurs, along with a remarkable increase of emission intensity (see the Inset of Fig. 2b). The evolution of peak position and linewidth versus pump fluences are shown in Fig. S6, indicating that the blueshift (≤0.5 nm) appears while the linewidth becomes broader, which may be caused by several dynamical effects such as photoinduced thermal effect, optical density fluctuations, band filling, or electron-hole many-body interactions^38^. Through Gaussian function fitting analysis, the linewidth of individual lasing peak (*δλ*) near the threshold can be identified as 0.23 nm, and the cavity quality factor *Q* = *λ/δλ* of as-grown quasi-2D nanolasers in our study is ~2340 (Fig. 2d), which is higher than the previous report for (BA)_2_FA_*n*−1_Pb_*n*_Br_3*n*+1_ quasi-2D perovskite nanolasers^39^.

### Double-pulsed lasing characteristics

**Fig. 3:**
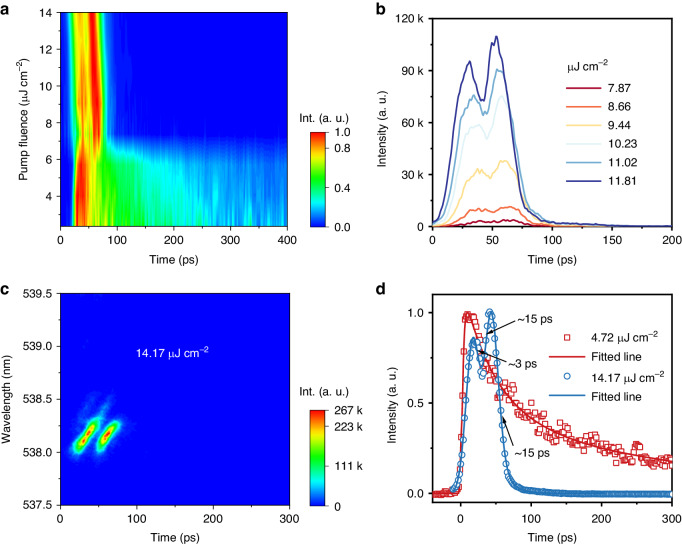
Double-pulsed lasing characteristics by TRPL measurements. **a** 2D pseudo-color plot of TRPL intensity versus different pump fluences from quasi-2D perovskite nanowires. **b** TRPL decay curves of quasi-2D perovskite nanowires pumped above the lasing threshold. **c** 2D pseudo-color TRPL image of double-pulsed lasing emission from quasi-2D perovskite nanowires at the pump fluence of 14.17 μJ cm^−2^. **d** TRPL decay curves and their fitted lines of quasi-2D perovskite nanowires pumped before the lasing threshold (4.72 μJ cm^−2^) and after the lasing threshold (14.17 μJ cm^−2^)

To gain further insight into StE dynamics in the gain media, TRPL is measured by using a streak camera with a time resolution of less than 1 ps. And intriguingly, the double-pulsed characteristics can be observed from the streak camera. Figure 3a clearly shows the evolution of double pulse with the increasing of the pump fluences above the lasing threshold. Two separated transient stimulated pulses appear with a time interval of around 22 ps as reflected by two peaks in decay curves (Fig. 3b) and two bright spots in streak camera images (Fig. 3c). Through exponential fitting analysis, the decay lifetime of the second pulse is 15 ps (Fig. 3d), which is much shorter than that of the spontaneous emission before the lasing threshold. We also conduct a comparison between quasi-2D nanowire and 3D nanowire lasing, as shown in Fig. S7. In contrast, only one simulated pulse is observed from the 3D samples under exactly the same experimental conditions. For such an optical gain medium with homogeneous dimensionality, the photoexcited charge carriers are directly injected into the optical gain excited levels within laser pulse duration, and then the excited hot carriers relax rapidly to the band edge via carrier–photon interaction, followed by the recombination^8^.

Combined with the previous nanowire structure characterization, we consider that the observation of the double-pulsed lasing emission from 2D nanowires after a single 400 nm femtosecond laser pulse pumping can be attributed to the multistep energy funneling process manifested as one fast process from the adjacent small-*n* QWs to large-*n* QWs (active gain region) and another slow process from the spatially separated ones. The energy funneling from spatially separated “energy storage” levels to the gain transitions provides the essential conditions of double-pulsed StEs. Generally, the intrinsic excitation of 3D-like perovskites and the subsequent energy transfer from low-dimensional phases may lead to the emergence of dual laser pulses in our case. We then examine the streak camera normalized image of double-pulsed lasing emission with respect to the wavelength at the pump fluence of 14.17 μJ cm^−2^ (Fig. S8). More surprisingly, it can be found that there has been suspicion of the existence of another pulse at the long-wavelength side of the first pulse. This finding prefigures that the quasi-2D perovskite nanowires have great potential for developing triple-pulse lasers. The further double-pulsed lasing mechanism is discussed in detail as follows.

### Double-pulsed lasing mechanism

To the best of our knowledge, the luminescence behaviors, along with carrier density, are governed by the basic carrier decay physics, which is primarily determined by the structural dimension of the perovskite rather than the materials’ composition^40^. Therefore, it is imperative to understand the excited carriers' decay channels in various dimensional perovskites to elucidate the generation of double-pulsed lasing. The exciton–phonon interactions are hence taken into consideration to estimate the carrier decay dynamics in various phases because the phonon interaction affects the charge-carrier mobility and hot carrier cooling process, and it makes a critical difference in the linewidth and shape of the perovskite emission^41^. Note that, in order to analyze exciton–phonon interactions, exciton transfer and carrier recombination dynamics, we take quasi-2D perovskite thin films as the samples in the following section, which possess analogous dimensional phase compositions as nanowires. The comparison of the UV–vis absorption spectrum between quasi-2D perovskite nanowires and thin films is shown in Fig. S9.

**Fig. 4:**
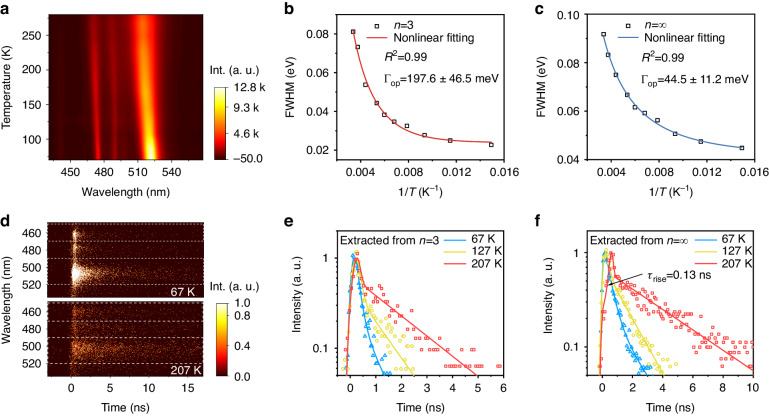
Exciton–phonon coupling in quasi-2D perovskite thin films. **a** 2D pseudo-color plot of PL intensity versus different temperatures. **b** and **c** FWHMs extracted from the PL spectra versus temperature for *n* = 3 and *n* = ∞, respectively. **d** 2D pseudo-color TRPL images from quasi-2D perovskite thin films at the temperature of 67 and 207 K from the top to bottom panels, respectively. **e** and **f** TRPL decay curves and their fitted lines of quasi-2D perovskite thin films at the temperature of 67, 127 and 207 K for *n* = 3 and *n* = ∞, respectively

To quantitatively determine the exciton–phonon coupling strength in MQW structures, temperature-dependent PL spectroscopy was performed (Figs. 4a and S10). Although the dominant component in the perovskite film is *n* = 2, the emission peak of this ultralow dimensional phase can hardly be observed under low pump fluence, resulting from the efficient energy transfer. All observed PL peaks at different locations gradually become broadening and more asymmetric with increasing temperature. The broadened and asymmetrical PL emission at high temperatures (>180 K) generally arises from the phonon scattering and self-trapped excitons emission (at low photon energy)^42^. And the exciton–phonon interaction is believed the dominant factor for broadening at low injected carrier density^43^. The FWHM of PL emissions at different emission positions (~2.35 eV corresponding to *n* = ∞, and 2.63 eV corresponding to *n* = 3) as a function of temperature was extracted as presented in Fig. 4b, c, which is well described with the phenomenological model of Γ(*T*) = Γ_0_ + Γ_LO_ (see Supplementary Note 1) with Γ_LO_ for *n* = ∞ and *n* = 3 are 44.5 ± 11.2 and 197.6 ± 46.5 meV, respectively (Table 1). The exciton–phonon coupling strength (Γ_LO_ = 197.6 meV) for *n* = 3 QWs is comparable with the values of *n* = 3 component (Γ_LO_ ~ 250 meV) in the previous report^44^, whereas is much larger than the *n* = 3 pure phase thin flake (Γ_LO_ = 70 meV) in the previous work^45^, which may due to the lateral confinement of the exciton in the nano-sized octahedral layer in the perovskite MQWs. The exciton–phonon coupling data in the references can be seen in Table S1. The exciton–phonon interaction of low *n* phases (*n* = 3) is much higher than that of *n* = ∞ phase (Γ_LO_ = 44.5 meV) in this perovskite, suggesting that the crystal lattice rigidity always reduces with the decreasing of inorganic layer thickness^46^.

**Table 1:** Fitting result of temperature versus FWHM of the PL spectra

Components	Γ_0_^a^ [meV]	Γ_LO_^b^ [meV]	*E*_LO_^c^ [meV]
*n* = ∞	42.3 ± 1.6	44.5 ± 11.4	16.7 ± 3.4
*n* = 3	23.9 ± 1.4	197.6 ± 46.5	38.1 ± 4.8

^a^Γ_0_ is the inhomogeneous broadening factor

^b^Γ_LO_ is exciton–longitudinal optical (LO) phonon coupling strength

^c^*E*_LO_ = *ħϖ* is the LO-phonon energy

It is noteworthy that small-*n*-dominated perovskites are not favorable for achieving lasing emission owing to strong electron–phonon interactions with faster band edge-to-trap process and the lack of optical gain from biexcitons^47–49^. However, strong electron–phonon interactions may play a critical role in the laser pulse multiplication in our case. The relatively high electron–phonon coupling strength in the lower *n* phases can slow-down the carrier mobility, therefore prolonging the carrier funneling process from lower dimensional phases to the higher counterparts (*n* = ∞), which provides essential conditions for the generation of the double laser pulse. Furthermore, the weaker phonon scattering in *n* = ∞ phase may contribute to the optical gain formation for low threshold laser, and the continuous exciton localization from stacked mixed low-dimensional phases to the higher dimensional phases (acting as gain active region) may play a key role in the generation of double-pulsed lasing. The relative exciton–phonon coupling strength in those two phases can also be extracted from the temperature-dependent PL band gap energy (see Fig. S11, Table S2 and Supplementary Note 2), which is exactly the same as that in the above fitting results.

To corroborate the photophysical process in the multiple dimensional perovskites, the temperature-dependent TRPL measurements were pursued to deeply understand the mechanisms of how the exciton–phonon interactions affect the energy transfer process. We used the 400 nm, 100 kHz pump laser to excite the mixed-dimensional perovskite films. The temperature-dependent TRPL curves were collected with a wide time window that simultaneously covering the signals of low-dimensional phases (*n* = 3–5) and high-dimensional phase (*n* = ∞), allowing us to pursue the evolution of the energy transfer process and conclude the influence of exciton–phonon interactions in the transfer process. We observed that the emission signals of low-dimensional phases of *n* = 3 and high-dimensional phases of *n* = ∞ are individual and clear enough to analyze, therefore, the TRPL signals of *n* = 3 and *n* = ∞ at various temperatures were extracted, respectively, for the further demonstration (Figs. 4d–f and S12). At low temperatures, both two-dimensional phases exhibit a bi-exponential decay. As the rising of temperature, the lifetime of exciton recombination in both *n* = 3 and *n* = ∞ gradually gets longer, which can be attributed to the thermally activated phonon scattering. The self-trapped exciton will not fully result in nonradiative recombination; the trapped excitons near the band edge possibly transform into free excitons under the strong phonon vibration, thereby inducing a prolonged fluorescence lifetime^50^. It should be noted that the decay curve of *n* = ∞ phase starts to show an initial building-up process at high temperature, indicating a slow-down energy transfer rate from low-dimensional to high-dimensional phases. The enhanced electron–phonon coupling strength at high temperatures, especially in low-dimensional phases, is believed to be a primary motivator for this slow-down charge-carrier transfer process. The prolonged carrier building-up process in gain medium (large-*n* QWs phases) provides an essential condition for the generation of double-pulsed lasing. Therefore, the stepped strengthen phonon scatterings in perovskite MQWs are considered to be a vital origin for the generation of double-pulsed lasing.

**Fig. 5:**
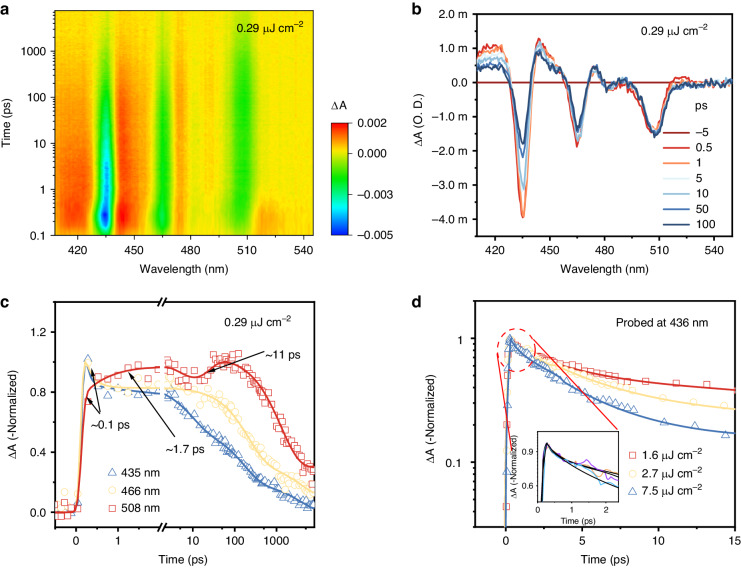
Ultrafast carrier transfer and recombination dynamics in quasi-2D perovskite thin films by TA measurements. **a** 2D pseudo-color plot of the obtained broadband TA spectra in between 50 fs to 7600 ps subsequent to 400 nm excitation at the pump fluence of 0.29 μJ cm^−2^. **b** TA spectra at selected timescales under pump fluence of 0.29 μJ cm^−2^. **c** TA kinetics traces probed at different wavelengths of 435, 466 and 508 nm, respectively. **d** TA kinetics probed at the excitonic photobleaching peak (at 436 nm) of low-dimensional (*n* = 2) QWs under different pump fluences

To further investigate the transfer and recombination dynamics of photogenerated carriers in our mixed-dimensional perovskite, the femtosecond transient absorption (TA) spectra were measured under various pump fluences. As shown in the TA spectra (Fig. 5a, b), several distinct photobleaching (PB) peaks were observed, originating from the state filling caused by the photon-excited excitons in various dimensional phases (*n* = 2, 3, 4 and ∞), which is well-matched with the steady-state absorption spectra. The time evolution of TA signals at different exciton resonance peaks reveals the carrier localization dynamics from low-dimensional phase to 3D-like counterparts. The kinetics curves of these exciton resonances were then extracted, as shown in Fig. 5c. We found that the PB signatures at *n* = 2 and *n* = 3 exciton resonance appear instantly within 150 fs (almost consistent with laser pulse), which confirms that the excitons are firstly created in this low-dimensional perovskite layer. Following the fast building-up process, the PB at *n* = 2 and *n* = 3 exciton resonance show an ultrafast decay with a time constant of 0.1 ps. Meanwhile, a rising process with a lifetime close to this fast decay process was observed at the PB of *n* = ∞ perovskite layer, which can be assigned to the fast exciton transfer from *n* = 2 or *n* = 3 to the adjacent active layer. Following this fast exciton localization, there is a relatively slow rising process with a lifetime of a few picoseconds, which can be attributed to another exciton transfer process from the non-closely stacked low-dimensional component. It should be worth noting that there is a slight decay straight after the relatively slow rising process, indicating the recombination rate of charge carriers is faster than the rate of injection in the *n* = ∞ phases in this stage. Subsequently, one more rising process was observed, which may be ascribed to further carrier transfer process from the low-dimensional phase in the distance to the 3D-like phases. These observations indicate that there is unremitting carrier injection in high-dimensional perovskites accompanied by carrier recombination. The duration time of carrier injection to the high-dimensional component is around 60 ps when the injected carrier density is below 10^16^ cm^−3^ (Fig. S13). Such a continuous carrier injection process enables a positive increase of carrier concentration in high-dimensional phases. Thereby, it is sufficient to generate multiple-pulse lasing within 60 ps if the pulse interval is narrow enough. The pulse interval corresponds to energy transfer time in this material system, which may be tuned by modulating the organic cation spacers. These contents remain an open question and are beyond the scope of this work.

**Fig. 6:**
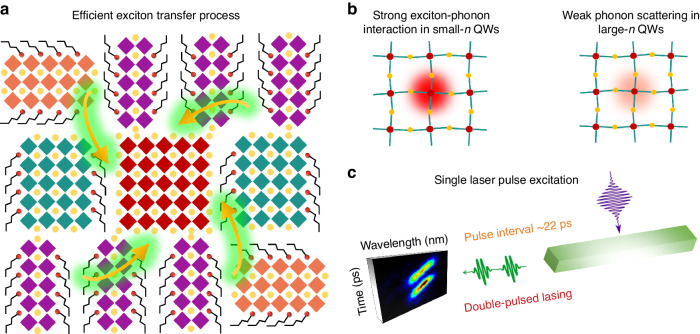
Schematic of double-pulsed lasing mechanism in quasi-2D perovskite nanowire. **a** Exciton localization from small-*n* QWs to large-*n* QWs. **b** Exciton–phonon interaction in different dimensional QWs. **c** Under 400 nm femtosecond single laser pulse excitation, double-pulsed lasing with a time interval of 22 ps is achieved in 2D nanowire

With the increase of the injected carrier density, population saturation of the excited states in *n* = ∞ phase blocks the exciton localization process and accelerates the rising time. Nonetheless, the rising process would persist at high pump fluence (near the lasing threshold) (Figs. 5d, S13 and S14). Based on the above TA dynamic findings, the double-pulsed lasing mechanism can be described as follows (Fig. 6). The dual laser pulses in our case originate from two efficient carrier funneling processes into the optical gain components: one fast energy transfer from the adjacent small-*n* QWs and another slow process from the spatially separated ones. Combined with the exciton–phonon coupling analysis, weak phonon scattering in the gain active region is favorable for the building-up of population inversion for low threshold lasing. Moreover, the carrier funneling into the gain region will be prolonged due to strong phonon scattering in low-dimensional components, leading to the emergence of the second pulse.

## Discussion

In summary, we achieved low threshold single-mode double-pulsed nanolasers using quasi-2D BA_2_Cs_*n*−1_Pb_*n*_Br_3*n*+1_ perovskites. The prepared nanowires exhibit excellent single-mode lasing emission at ~539 nm and a low threshold of 7.87 μJ cm^−2^, which benefits from the high *Q* (~2340) and low optical loss cavity. Through streak camera direct observations, the nanolasers exhibit double-pulsed lasing characteristics with a pulse width of 28 ps and time interval of 22 ps. We demonstrated that the double-pulsed StEs, in our case, originate from one fast energy transfer from the adjacent small-*n* QWs and another slow process from the spatially separated ones to the gain active region, respectively. Furthermore, strong electron–phonon coupling in small-*n* QWs delays the carrier funneling process, while weak phonon scattering in large-*n* QWs permits continuous exciton localization, enabling population inversion doubling. We anticipate that this work paves the way towards the realization of double or multiple-pulse perovskite lasers.

## Materials and methods

### Materials

All reagents were used without further purification: cesium bromide (CsBr, 99.999%), lead bromide (PbBr_2_, 99.999%), butylammonium bromide (BABr, 99.5%), *N*,*N*-dimethylformamide (DMF, ≥99.5%), ammonium bromide (NH_4_Br, 99.998%), hexane (GR, ≥99.5%), acetone (AR, ≥99.5%), and ethanol (AR, ≥99.7%).

### Synthesis of quasi-2D perovskite nanowires

For the synthesis of BA_2_Cs_*n*−1_Pb_*n*_Br_3*n*+1_ quasi-2D perovskite nanowires, 1.1 g PbBr_2_ was dissolved completely with constant stirring in 10 ml DMF at 60 °C. Then, 0.85 g CsBr was added to the PbBr_2_ solutions until completely dissolved. Finally, 0.47 g BABr was dissolved in the above solution with constant stirring for 2 h. The resulting precursor solutions are spin-coated onto the substrates at 4500 rpm for 60 s and annealed to accelerate crystallization at 100 °C for 5 min. All the preparation processes are carried out in the air atmosphere.

### Synthesis of quasi-2D perovskite thin films

The precursors of PbBr_2_, CsBr, BABr and NH_4_Br with a molar ratio of 3:2:2:0.3 are dissolved in a DMSO solution with a Pb^2+^ molar concentration of 0.3 M. The solution is stirred at 60 °C for 1 h in a nitrogen environment. Quasi-2D perovskite thin films are deposited by spin coating this solution on the substrates. Then, the films are annealed at 100 °C for 10 min.

### Morphology and structure measurements

The morphologies and element analysis of the quasi-2D perovskite nanowires are examined using scanning electron microscopy with an energy-dispersive X-ray spectroscopy system (Hitachi S-4300). The ultraviolet–visible (UV–vis) absorption spectra are performed by a Cary 5000 UV–vis–NIR spectrometer.

### Time-resolved photoluminescence measurements

The lasing emission and TRPL properties of the quasi-2D perovskite nanowire are performed with a home-built femtosecond pulsed laser system. The second harmonic (400 nm, 50 fs, 1 kHz) of a regenerative amplifier seeded with a mode-locked Ti:sapphire laser (Verdi G8, Coherent, America) was focused vertically onto the samples. The excitation light was focused using a lens to form a circular facula with a diameter of 20 μm. The lasing emission from the samples was vertically collected and detected with a multi-channel cooled charge-coupled detector (CCD) and spectrally resolved by a triple-grating spectrometer. After collection, the lasing emission was directed into the streak camera system (C10910, Hamamatsu, Japan) to investigate the time-resolved kinetics. Note that all the optical measurements were carried out with samples in ambient air at room temperature.

### Femtosecond transient absorption measurements

The broadband femtosecond TA spectra were collected by using a TA spectrometer (HELIOS, Ultrafast System). The white light probe beam (420–800 nm) was generated by focusing a small portion (around 10 μJ) of the fundamental 800 nm laser pulses into a 2 mm sapphire plate. The 400 nm pump pulses were obtained by doubling the fundamental 800 nm pulses with a BBO crystal and focused on the sample with a beam size of 2 mm in diameter.

### Supplementary information

Supplementary Materials

## References

[1] Marinelli A (2015). High-intensity double-pulse X-ray free-electron laser. Nat. Commun..

[2] Krogen P (2017). Generation and multi-octave shaping of mid-infrared intense single-cycle pulses. Nat. Photonics.

[3] Mayer B (2017). Long-term mutual phase locking of picosecond pulse pairs generated by a semiconductor nanowire laser. Nat. Commun..

[4] Cowley J (2017). Excitation and control of plasma wakefields by multiple laser pulses. Phys. Rev. Lett..

[5] Wetzel B (2018). Customizing supercontinuum generation via on-chip adaptive temporal pulse-splitting. Nat. Commun..

[6] Shinohara Y (2020). Split-pulse X-ray photon correlation spectroscopy with seeded X-rays from X-ray laser to study atomic-level dynamics. Nat. Commun..

[7] Mao D (2021). Synchronized multi-wavelength soliton fiber laser via intracavity group delay modulation. Nat. Commun..

[8] Guo J (2020). Ultrashort laser pulse doubling by metal-halide perovskite multiple quantum wells. Nat. Commun..

[9] Qin CJ (2020). Stable room-temperature continuous-wave lasing in quasi-2D perovskite films. Nature.

[10] Wang XZ (2023). Quasi-2D Dion–Jacobson phase perovskites as a promising material platform for stable and high-performance lasers. Sci. Adv..

[11] Raghavan CM (2018). Low-threshold lasing from 2D homologous organic–inorganic hybrid Ruddlesden–Popper perovskite single crystals. Nano Lett..

[12] Alvarado-Leaños AL (2022). Lasing in two-dimensional tin perovskites. ACS Nano.

[13] Gao W (2022). Two-photon lasing from two-dimensional homologous Ruddlesden–Popper perovskite with giant nonlinear absorption and natural microcavities. ACS Nano.

[14] Li Y (2022). Lasing from laminated quasi-2D/3D perovskite planar heterostructures. Adv. Funct. Mater..

[15] Wang NN (2016). Perovskite light-emitting diodes based on solution-processed self-organized multiple quantum wells. Nat. Photonics.

[16] Yuan MJ (2016). Perovskite energy funnels for efficient light-emitting diodes. Nat. Nanotechnol..

[17] Fu YP (2019). Metal halide perovskite nanostructures for optoelectronic applications and the study of physical properties. Nat. Rev. Mater..

[18] Deng SB (2020). Long-range exciton transport and slow annihilation in two-dimensional hybrid perovskites. Nat. Commun..

[19] Zhang HH (2018). 2D Ruddlesden–Popper perovskites microring laser array. Adv. Mater..

[20] Li MJ (2018). Enhanced exciton and photon confinement in Ruddlesden–Popper perovskite microplatelets for highly stable low-threshold polarized lasing. Adv. Mater..

[21] Lei L (2020). Efficient energy funneling in quasi-2D perovskites: from light emission to lasing. Adv. Mater..

[22] Pina JM (2021). Deep-blue perovskite single-mode lasing through efficient vapor-assisted chlorination. Adv. Mater..

[23] Liu ZZ (2021). Subwavelength-polarized quasi-two-dimensional perovskite single-mode nanolaser. ACS Nano.

[24] Li GH (2023). Localized bound multiexcitons in engineered quasi-2D perovskites grains at room temperature for efficient lasers. Adv. Mater..

[25] Bera KP (2023). Fabry–Perot oscillation and resonance energy transfer: mechanism for ultralow-threshold optically and electrically driven random laser in quasi-2D Ruddlesden–Popper perovskites. ACS Nano.

[26] Quan LN (2016). Ligand-stabilized reduced-dimensionality perovskites. J. Am. Chem. Soc..

[27] Kong LM (2021). Smoothing the energy transfer pathway in quasi-2D perovskite films using methanesulfonate leads to highly efficient light-emitting devices. Nat. Commun..

[28] Blancon JC (2018). Scaling law for excitons in 2D perovskite quantum wells. Nat. Commun..

[29] Cinquino M (2021). Managing growth and dimensionality of quasi 2D perovskite single-crystalline flakes for tunable excitons orientation. Adv. Mater..

[30] Lei YS (2022). Perovskite superlattices with efficient carrier dynamics. Nature.

[31] Gu H (2023). Phase-pure two-dimensional layered perovskite thin films. Nat. Rev. Mater..

[32] Ma DX (2021). Distribution control enables efficient reduced-dimensional perovskite LEDs. Nature.

[33] Wang K (2023). Suppressing phase disproportionation in quasi-2D perovskite light-emitting diodes. Nat. Commun..

[34] Zhu HM (2016). Screening in crystalline liquids protects energetic carriers in hybrid perovskites. Science.

[35] Becker MA (2018). Long exciton dephasing time and coherent phonon coupling in CsPbBr_2_Cl perovskite nanocrystals. Nano Lett..

[36] Straus DB (2016). Direct observation of electron–phonon coupling and slow vibrational relaxation in organic-inorganic hybrid perovskites. J. Am. Chem. Soc..

[37] Fu M (2018). Unraveling exciton–phonon coupling in individual FAPbI_3_ nanocrystals emitting near-infrared single photons. Nat. Commun..

[38] Zhu HM (2015). Lead halide perovskite nanowire lasers with low lasing thresholds and high quality factors. Nat. Mater..

[39] Zhang HH (2018). A two-dimensional Ruddlesden–Popper perovskite nanowire laser array based on ultrafast light-harvesting quantum wells. Angew. Chem. Int. Ed..

[40] Xing GC (2017). Transcending the slow bimolecular recombination in lead-halide perovskites for electroluminescence. Nat. Commun..

[41] Ni LM (2017). Real-time observation of exciton-phonon coupling dynamics in self-assembled hybrid perovskite quantum wells. ACS Nano.

[42] Thirumal K (2017). Morphology-independent stable white-light emission from self-assembled two-dimensional perovskites driven by strong exciton–phonon coupling to the organic framework. Chem. Mater..

[43] Lorke M (2006). Influence of carrier-carrier and carrier-phonon correlations on optical absorption and gain in quantum-dot systems. Phys. Rev. B.

[44] Long H (2019). Exciton–phonon interaction in quasi-two dimensional layered (PEA)_2_(CsPbBr_3_)_*n*−1_PbBr_4_ perovskite. Nanoscale.

[45] Liang Y (2019). Lasing from mechanically exfoliated 2D homologous Ruddlesden–Popper perovskite engineered by inorganic layer thickness. Adv. Mater..

[46] Gong XW (2018). Electron–phonon interaction in efficient perovskite blue emitters. Nat. Mater..

[47] Chong WK (2016). Dominant factors limiting the optical gain in layered two-dimensional halide perovskite thin films. Phys. Chem. Chem. Phys..

[48] Wang CH (2022). Low-threshold blue quasi-2D perovskite laser through domain distribution control. Nano Lett..

[49] Li YH (2023). Phase-pure 2D tin halide perovskite thin flakes for stable lasing. Sci. Adv..

[50] Peng SM (2020). Suppressing strong exciton–phonon coupling in blue perovskite nanoplatelet solids by binary systems. Angew. Chem. Int. Ed..

