# Supplementary material for: Pulse-doubling perovskite nanowire lasers enabled by phonon-assisted multistep energy funneling
Source: Light Sci Appl. 2024 Jul 17;13:170. doi: 10.1038/s41377-024-01494-2 (PMC11255266; doi:10.1038/s41377-024-01494-2)
Supplement: Supplementary file 1 — Supplementary Materials [file 41377_2024_1494_MOESM1_ESM.docx]

Supplementary Information for

Pulse-doubling perovskite nanowire lasers enabled by phonon-assisted multistep energy funneling

Chunhu Zhao^1^, Jia Guo^2^, Jiahua Tao^3🖂^, Junhao Chu^3^, Shaoqiang Chen^3🖂^ and Guichuan Xing^2🖂^

^1^Hunan Provincial Key Laboratory of Carbon Neutrality and Intelligent Energy, School of Resource & Environment, Hunan University of Technology and Business, Changsha 410205, China.

^2^Joint Key Laboratory of the Ministry of Education, Institute of Applied Physics and Materials Engineering, University of Macau, Macau 999078, China.

^3^Engineering Research Center for Nanophotonics and Advanced Instrument, Ministry of Education, School of Physics and Electronic Science, East China Normal University, Shanghai 200241, China.

Correspondence: Jiahua Tao (jhtao@phy.ecnu.edu.cn), Shaoqiang Chen (sqchen@ee.ecnu.edu.cn) or Guichuan Xing (gcxing@um.edu.mo)

These authors contributed equally: Chunhu Zhao, Jia Guo.


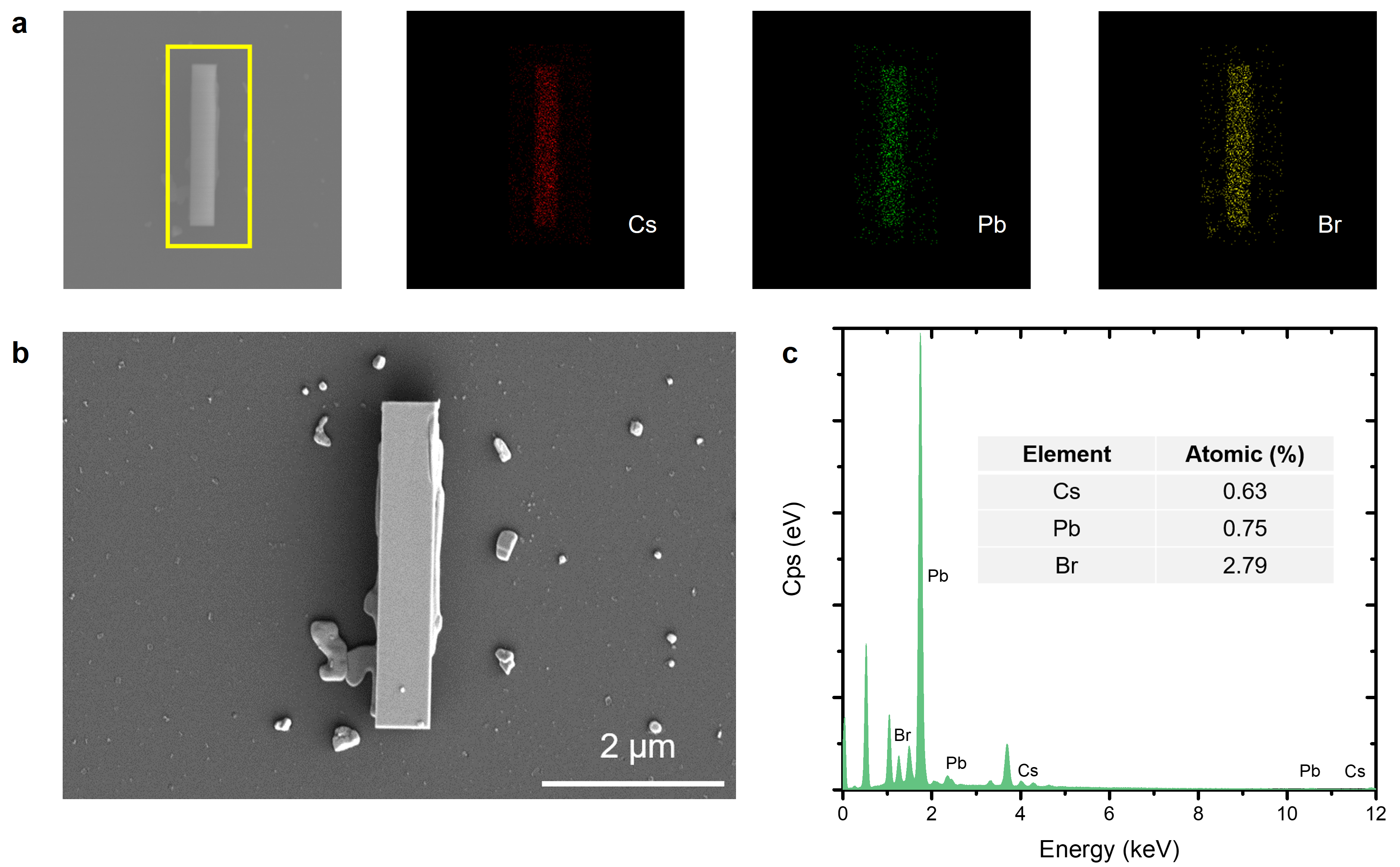


**Fig. S1 The EDS elemental mapping images of as-grown (BA)_2_Cs_n-1_Pb_n_Br_3n+1_ perovskite nanowire.** **a**, EDS elemental mapping of the nanowires. **b**, Corresponding SEM image of the single microwire on glass substrate; The scale bar is 2 μm. **c**, EDS spectra of the single nanowire; Inset: atomic ratios of Cs, Pb, and Br.


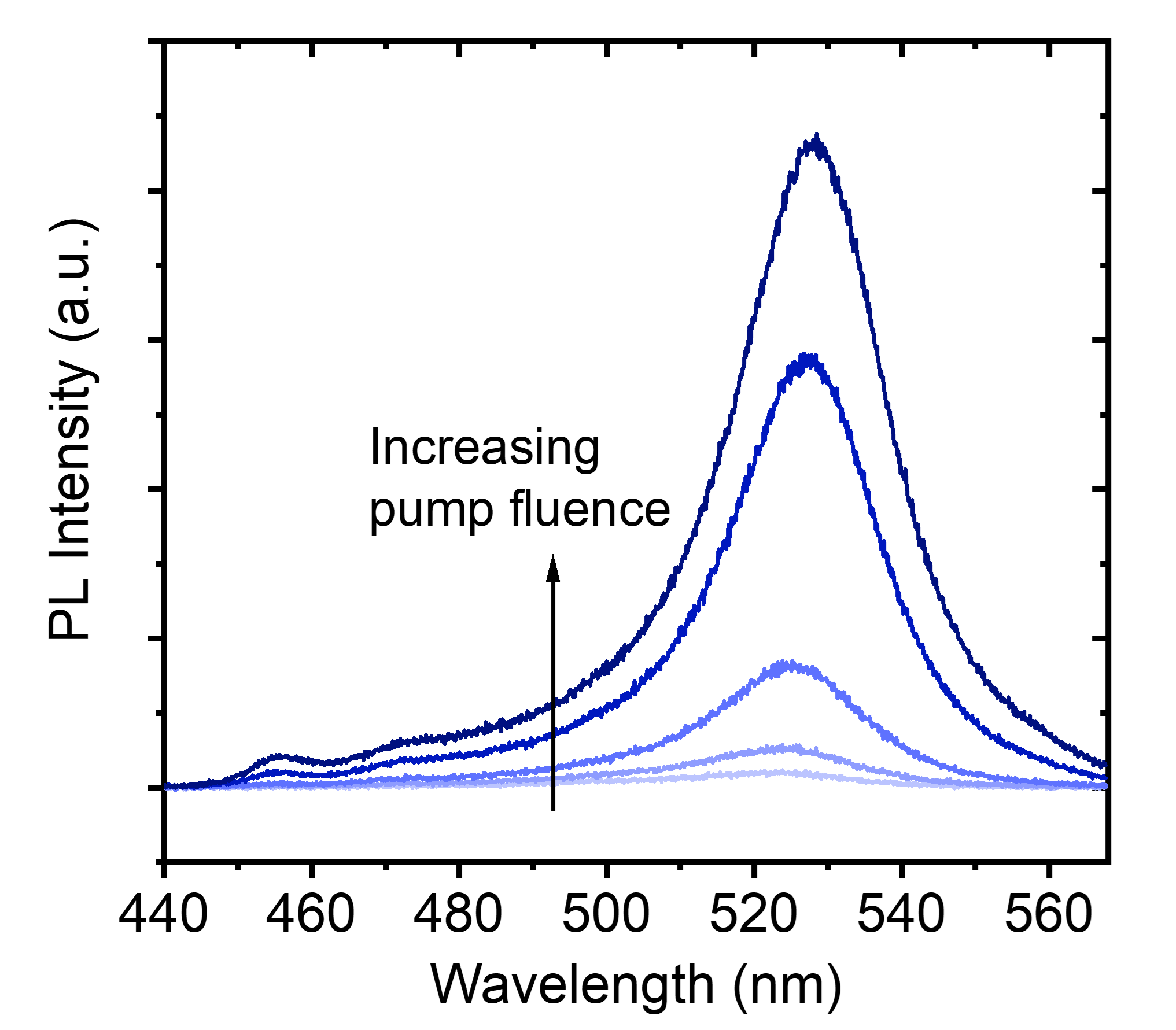


**Fig. S2 Photoluminescence (PL) spectra from quasi-2D perovskite nanowires.** The nanowires are excited under 400 nm femtosecond (fs) laser with the increase of pump fluence from 0.9 to 51 μJ cm^-2^.


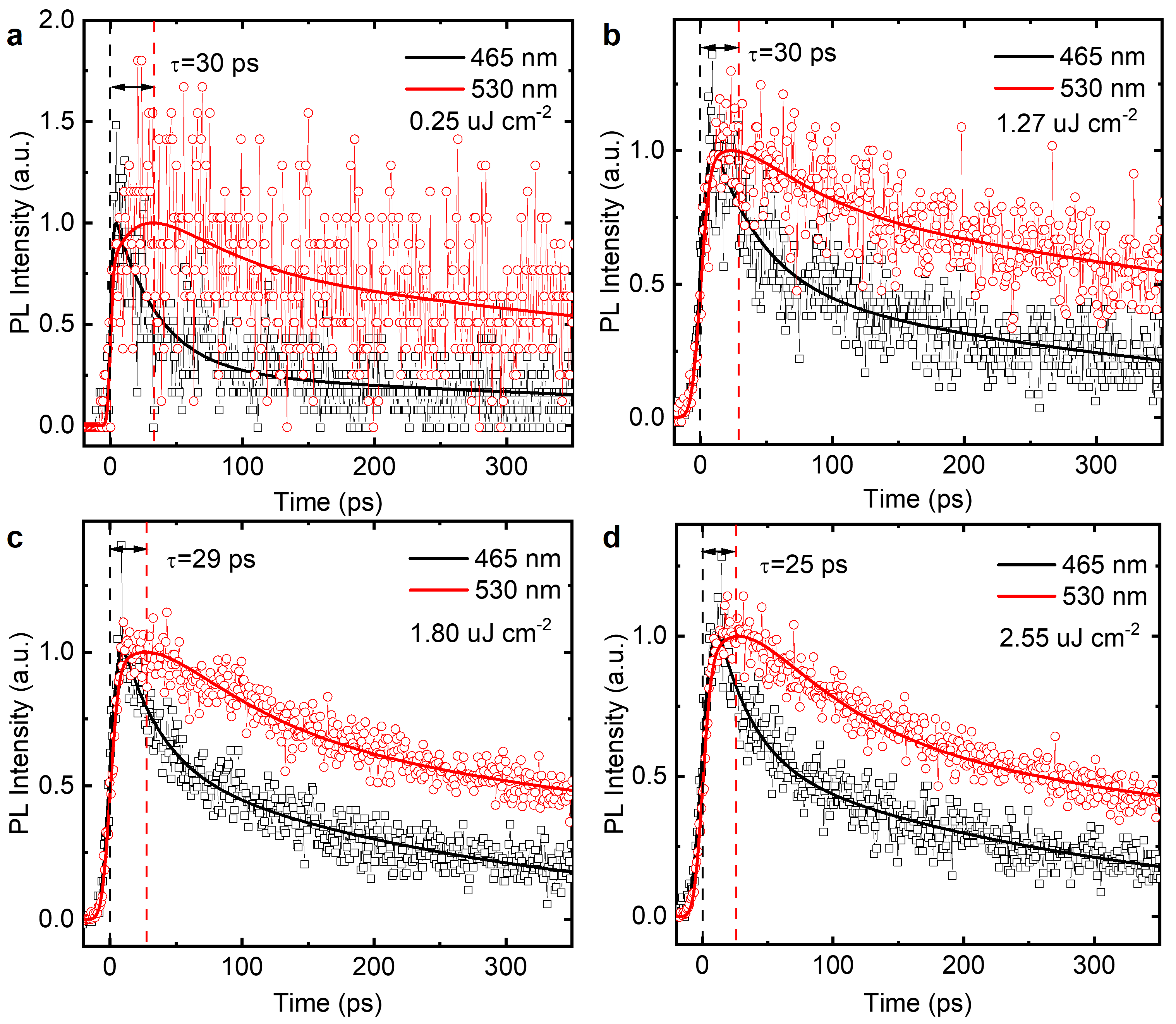


**Fig. S3 Time-resolved PL (TRPL) decay curves of a single nanowire under low injected carrier density. a**, **b**, **c** and **d** The single nanowire is excited by 400 nm, 50 fs, 1 kHz laser at the pump fluence of 0.25, 1.27, 1.80 and 2.55 μJ cm^-2^, respectively (the corresponding injected charge carrier density is 4.3×10^16^, 2.1×10^17^, 3.0×10^17^, 4.2×10^17^ cm^-3^).


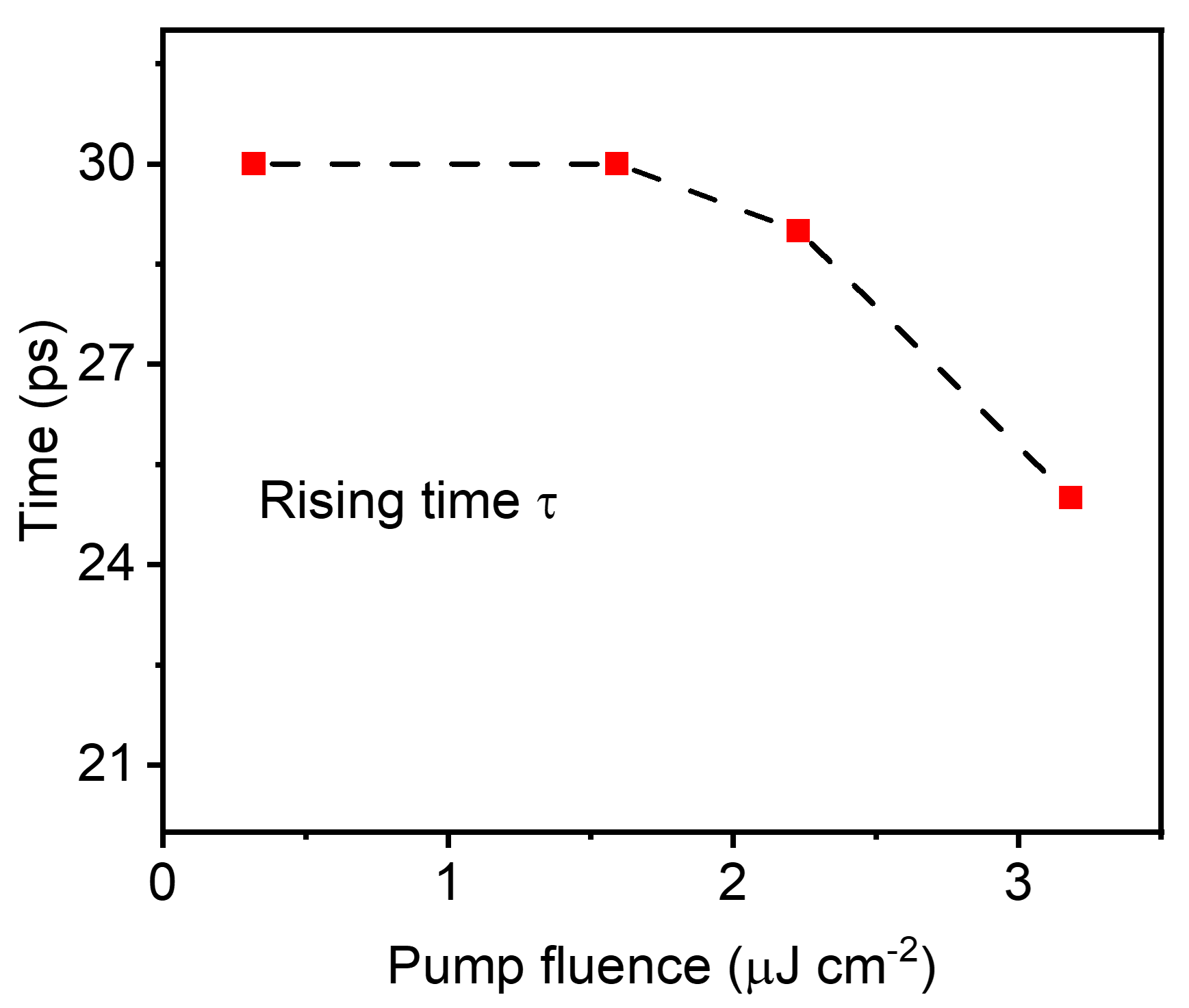


**Fig. S4 Lifetime of spontaneous emission at low pump fluence.** The lifetime of spontaneous emission remains at the same level of 30 ps when the pump fluence varies from 0.25 to 1.80 μJ cm^-2^. As the pump fluence increases to 2.55 μJ cm^-2^, the spontaneous emission shows fast lifetime and eventually approaches 25 ps. These features confirm the exciton transfer process in the nanowire.


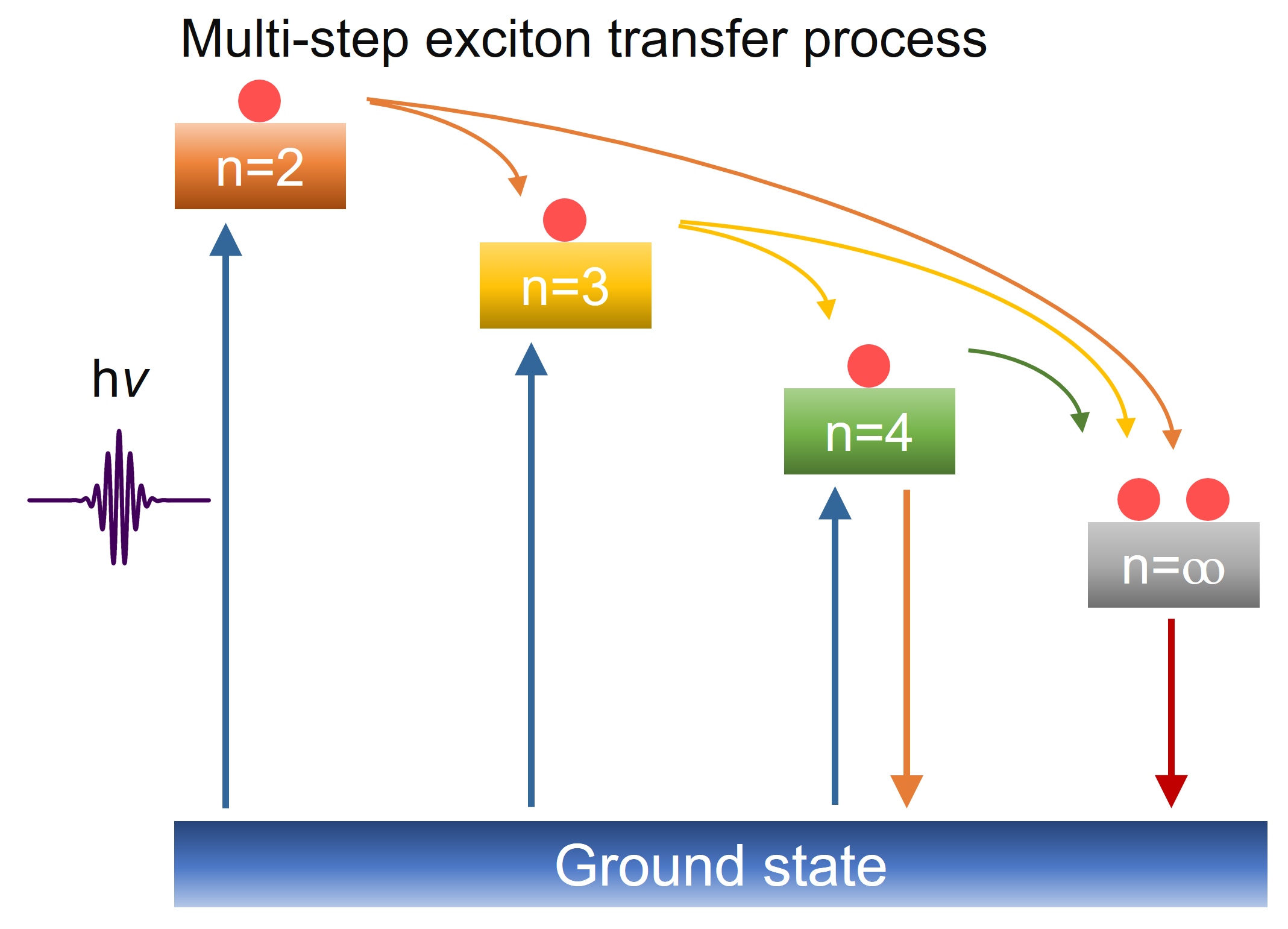


**Fig. S5 Schematic of cascade energy transfer process in quasi-2D perovskite nanowire.** Excitation energy is transferred downstream from smaller-*n* QWs to larger-*n* QWs, and the emission is mainly from larger-*n* QWs.


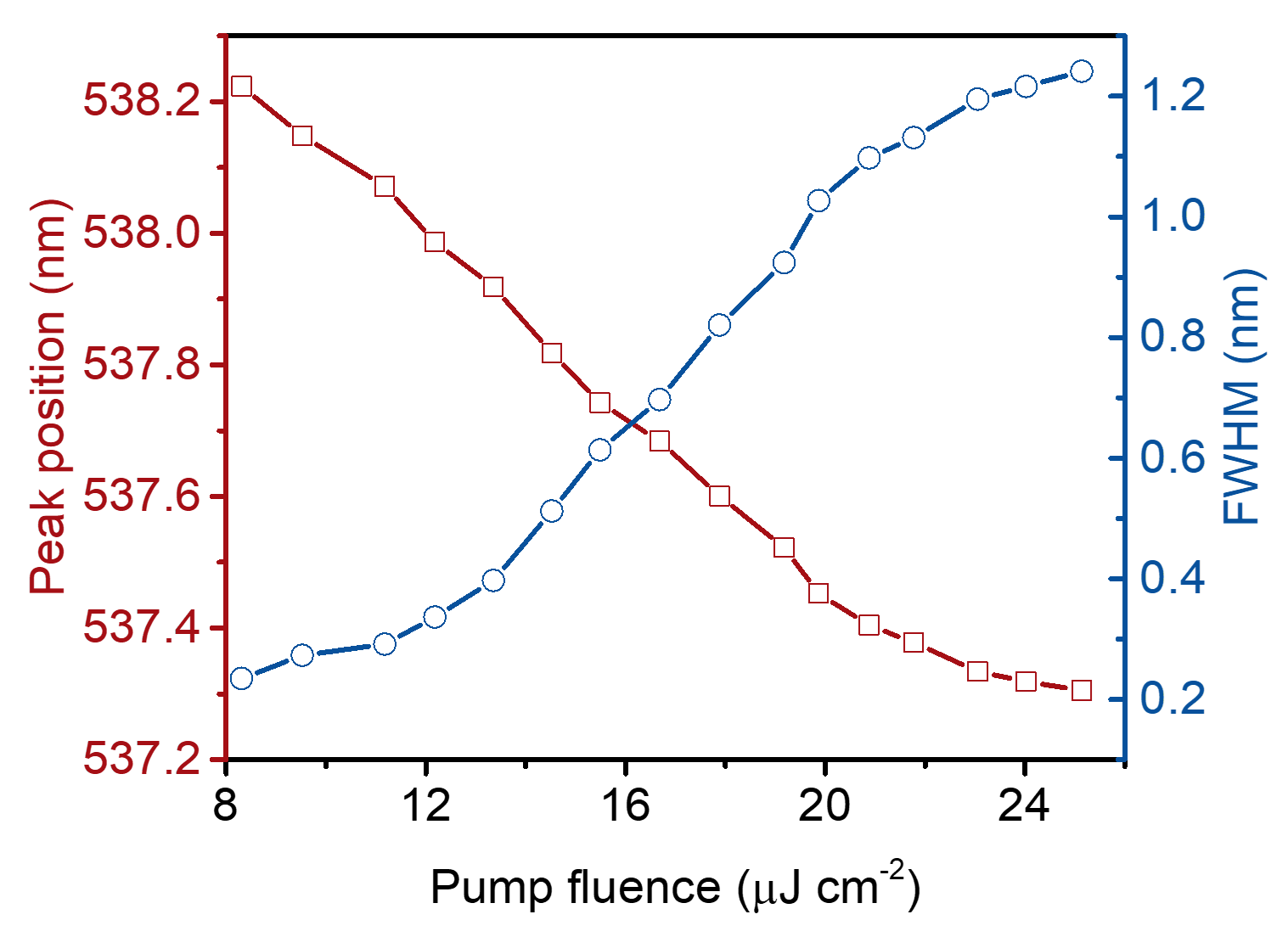


**Fig. S6 Evolution of peak position and FWHM versus pump fluences.** The peak position shows the blueshift of about 0.9 nm when the pump fluence increases from the lasing threshold of 7.87 μJ cm^-2^ to 25 μJ cm^-2^. And the line width gradually becomes broader.


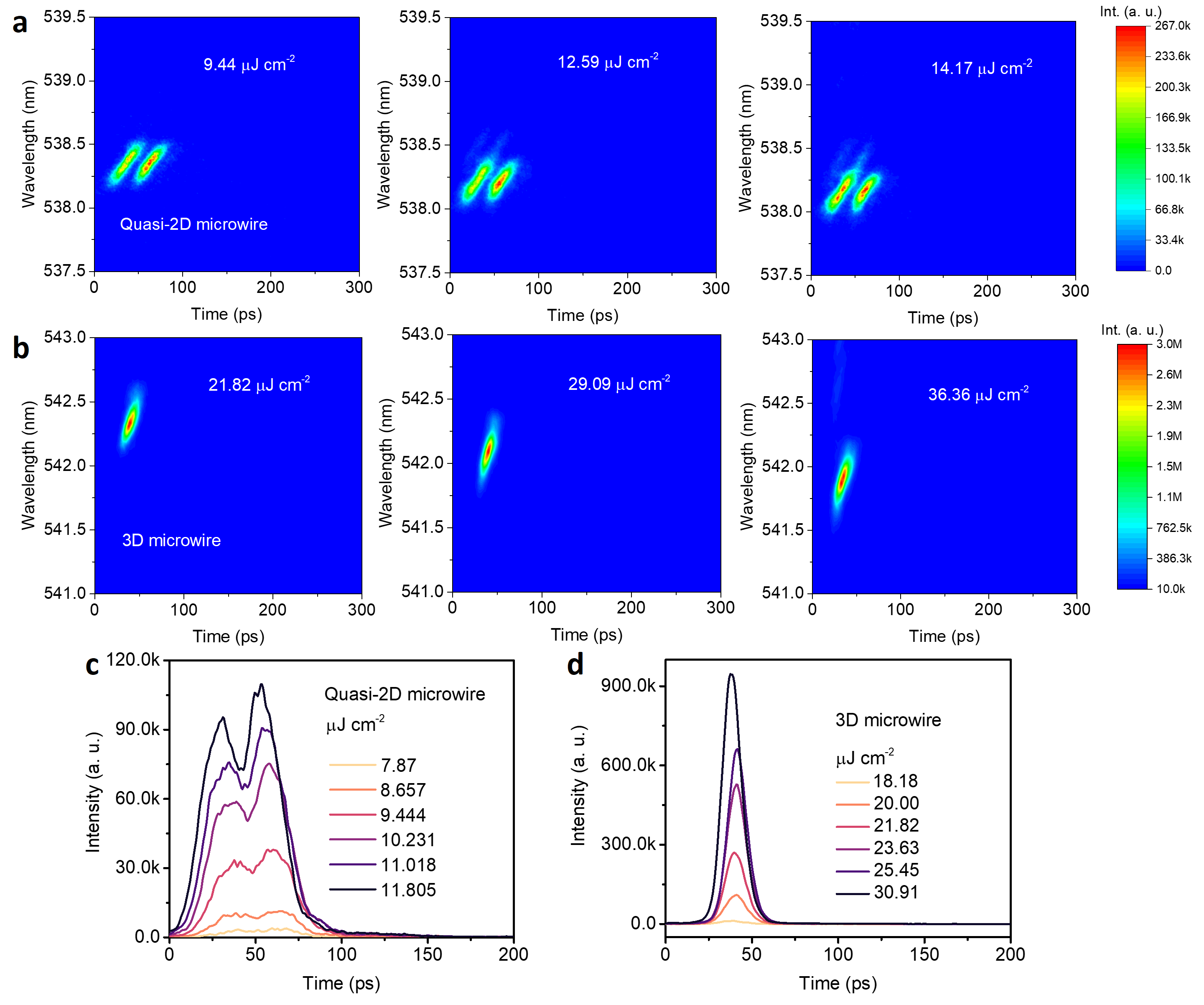


**Fig. S7 Double-pulsed lasing emission of quasi-2D perovskite nanowire in comparison with 3D perovskite nanowire.** **a**, 2D pseudo color plot of TRPL intensity above the lasing threshold from quasi-2D perovskite nanowire. **b**, 2D pseudo color plot of TRPL intensity above the lasing threshold from 3D perovskite nanowire. **c**, **d**, The extracted TRPL decay curves of quasi-2D and 3D perovskite nanowires pumped above the lasing threshold, respectively. These results were collected from samples excited with 400 nm laser pulses (1 kHz, 50 fs).


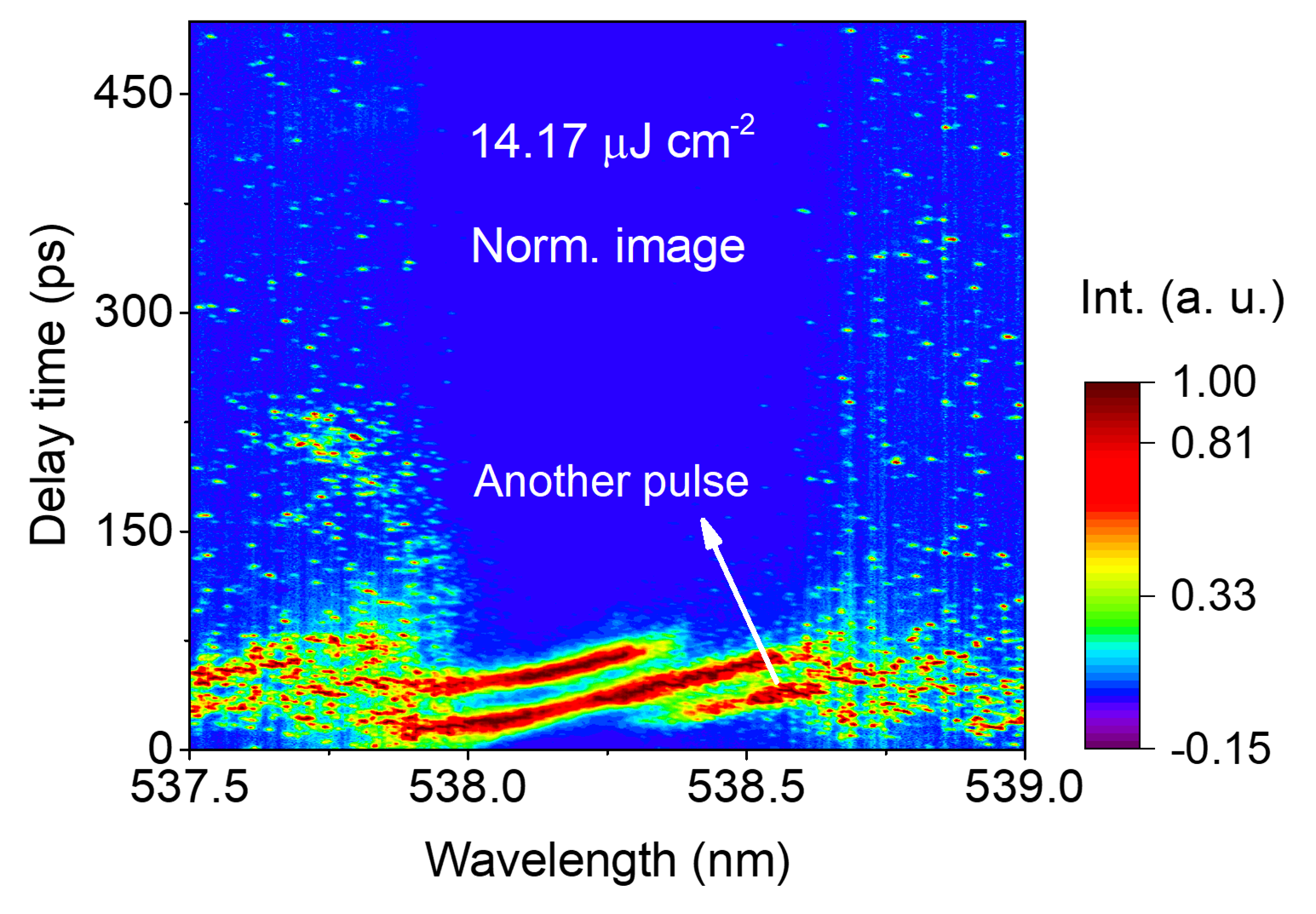


**Fig. S8 Normalized streak camera image of double-pulsed lasing emission at 14.17 μJ cm^-2^.** The TRPL image discloses a clear wavelength dependence of the delay time and pulse duration. Suspiciously, there has another pulse at the long-wavelength side of the first pulse.

**
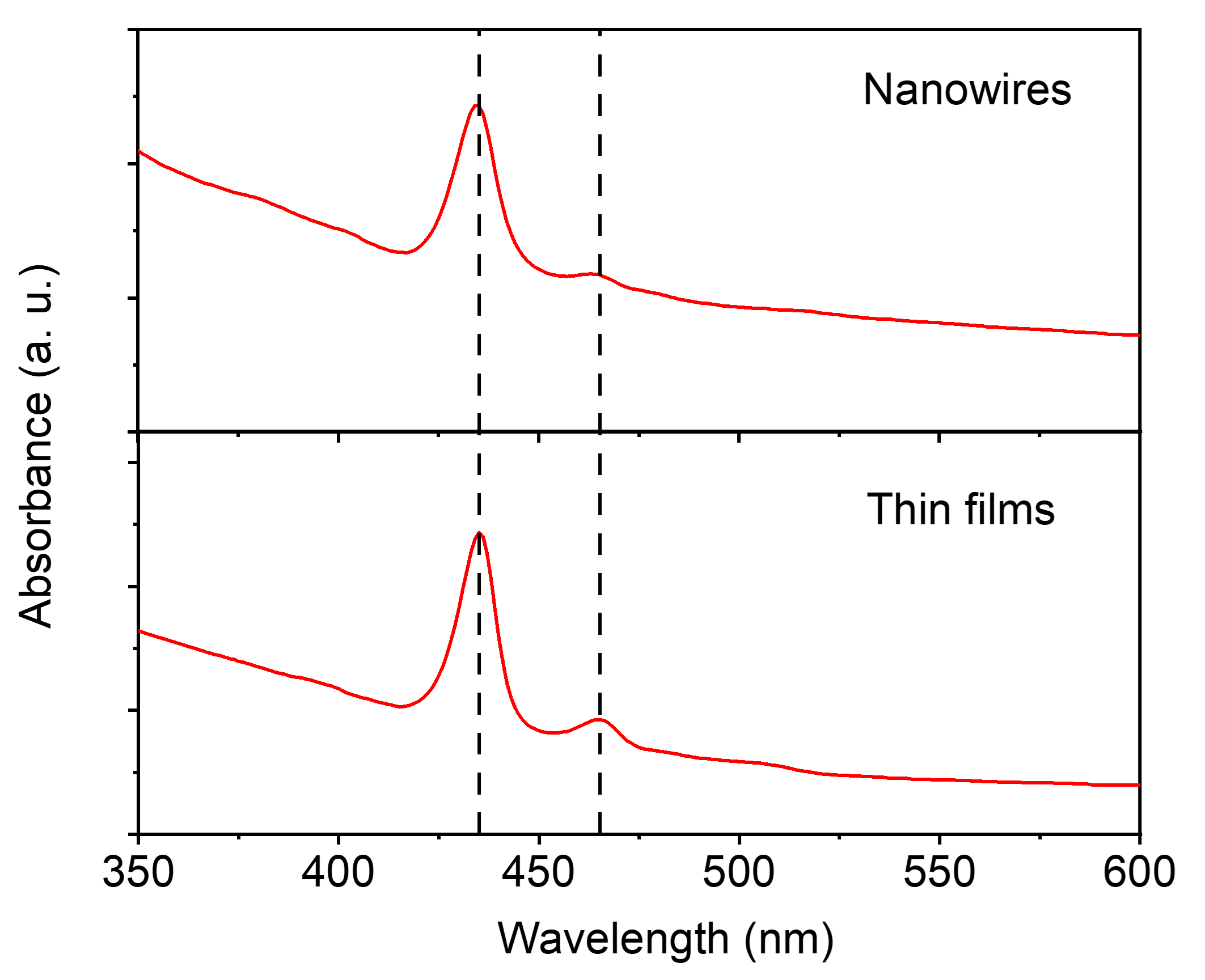
**

**Fig. S9 UV-vis absorption spectrum of 2D perovskite nanowires and thin films.** 2D perovskite thin films are employed as the samples to analyze the exciton-phonon interactions, exciton transfer process and carrier recombination dynamics in the following section. 2D perovskite thin films possess analogous dimensional phase compositions as nanowires.

**
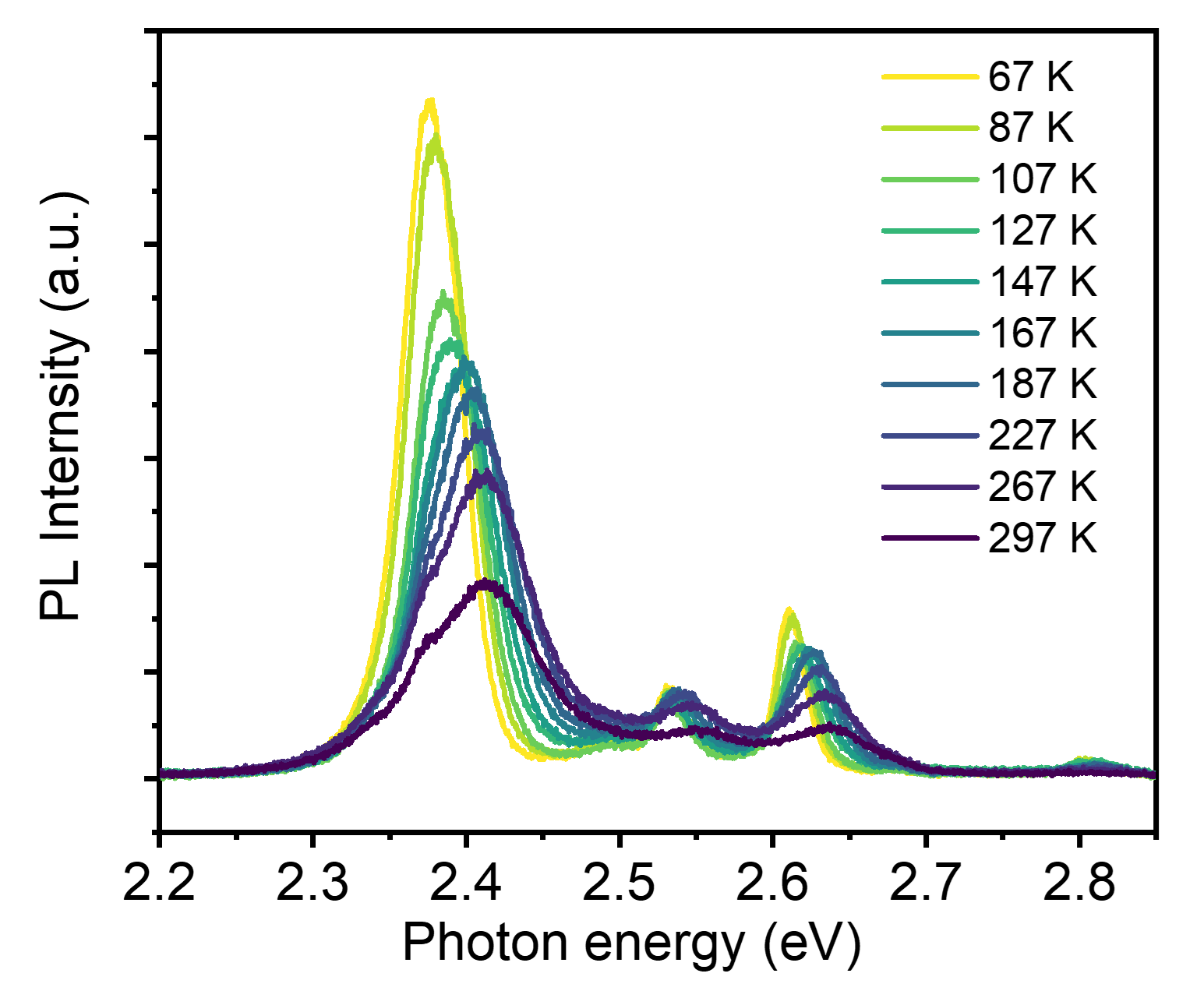
**

**Fig. S10 Typical PL spectra evolution of quasi-2D perovskite thin films with the temperature increase from 67 to 297 K.** The temperature values are indicated along with the PL spectra. As the temperature decreases, the blueshift of the PL spectra occurs from 2.41 eV to 2.37 eV.

**Table S1. Exciton-phonon coupling data in the references.**

| **Materials** | **Γ_0_ [meV]** | **Γ*_op_* [meV]** | **Γ*_LO_* [meV]** | **Reference** |
| --- | --- | --- | --- | --- |
| Nanoplate-2ML(CsPbBr_3_-OA-TOPO) | 27.4 | 186.5 | 43.4 | *Angew. Chem. Int. Ed.* **59**, 22156-22162 (2020) |
| Nanoplate-3ML | 43.4 | 120.3 | 29.0 | Ditto |
| Nanoplate-30%2ML70%3ML | 38.5 | 62.8 | 17.0 | Ditto |
| (PEA)_2_PbI_4_ | 30 | 70 | 29 | *J. Phys. Chem. Lett.* **10**, 13-19 (2019) |
| (PEA)_2_(CH_3_NH_3_)_2_Pb_3_I_10_ | 79 | 231 | 49 | Ditto |
| CH_3_NH_3_PbI_3-x_Cl_x_ | 38 | 92.1 | 25.3 | *Phys. Chem. Chem. Phys.* **16**, 22476-22481 (2014) |
| BA_2_Cs_n-1_Pb_n_Br_3n+1_ (n=∞) | 42 | 44 | 17 | Our work |
| BA_2_Cs_n-1_Pb_n_Br_3n+1_ (n=3) | 23 | 197 | 38 | Our work |

**
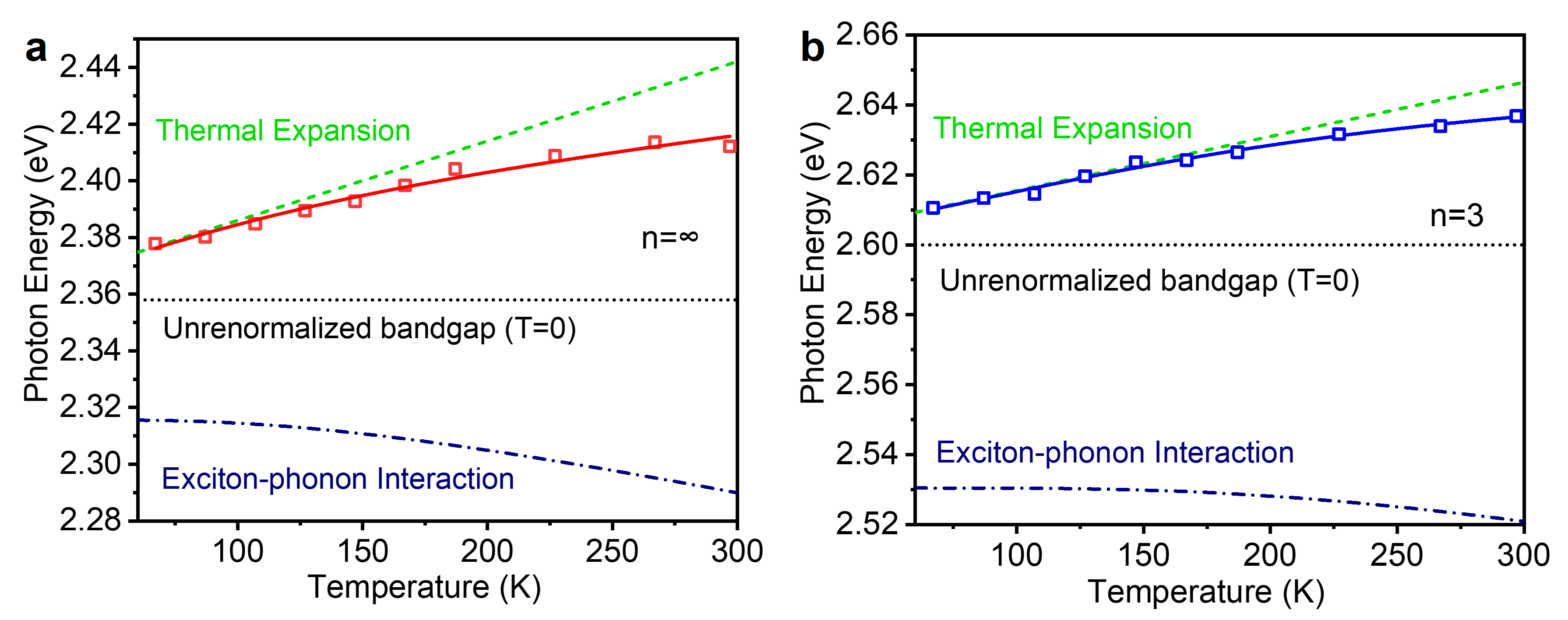
**

**Fig. S11 Fitting results of temperature dependent PL peak. a**, **b**, The solid fitting lines for *n*=∞ and *n*=3 phases, respectively. The dashed and the dashed-dotted line show the individual contributions of thermal expansion and exciton-phonon interaction.

**Table S2. Fitting result of temperature versus PL peak energy.**

|  | R^2 *^ | E_0_ [eV] | A _TE_ [meV] | A _EP_ [meV] | Θ [K] |
| --- | --- | --- | --- | --- | --- |
| ***n*=∞** | 0.975 | 2.40±0.06 | 0.28±0.2 | -42.41±40 | 438±73 |
| ***n*=3** | 0.99 | 2.67±0.12 | 0.15±0.3 | -69.61±11 | 823±61 |

* R^2^ is the goodness of fit.

**
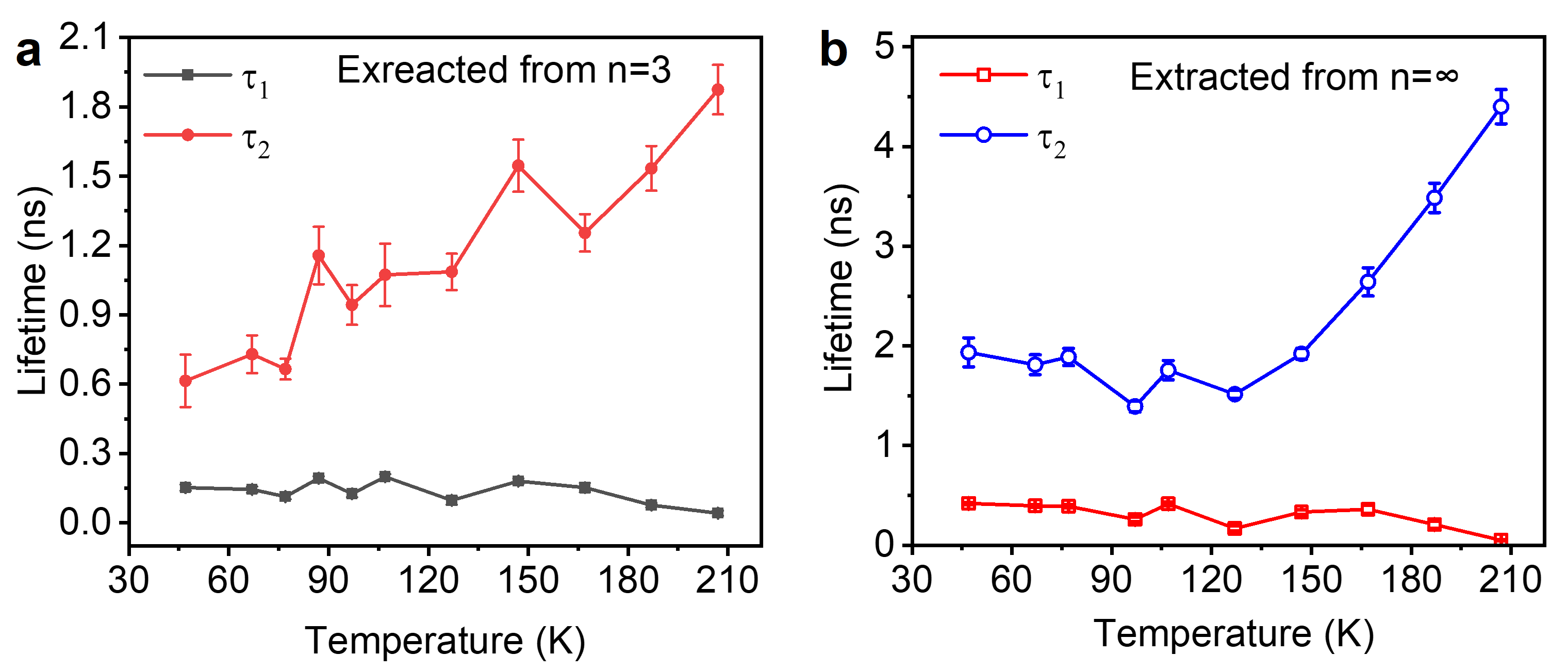
**

**Fig. S12 Lifetime versus temperature extracted from *n*=3 (a) and *n*=∞ (b) phases.** With the rising of temperature, the lifetime of exciton recombination in both *n*=3 and *n*=∞ gradually gets long, indicating the thermally activated phonon scattering.


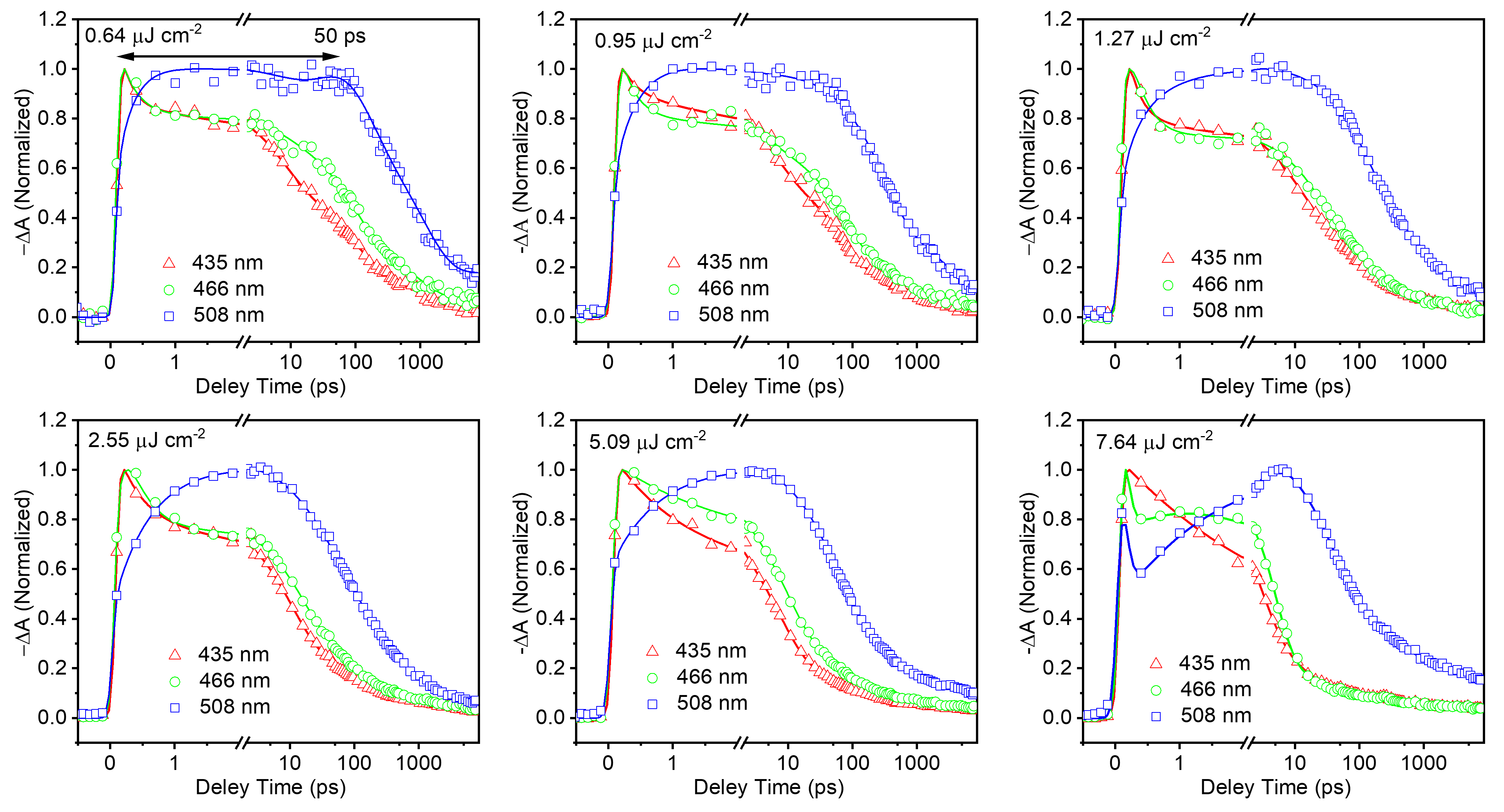


**Fig. S13 TA kinetics traces probed at different wavelengths under different pump fluences.** These TA spectrums elucidate multi-step carrier transfer process in quasi-2D perovskite thin films.

**
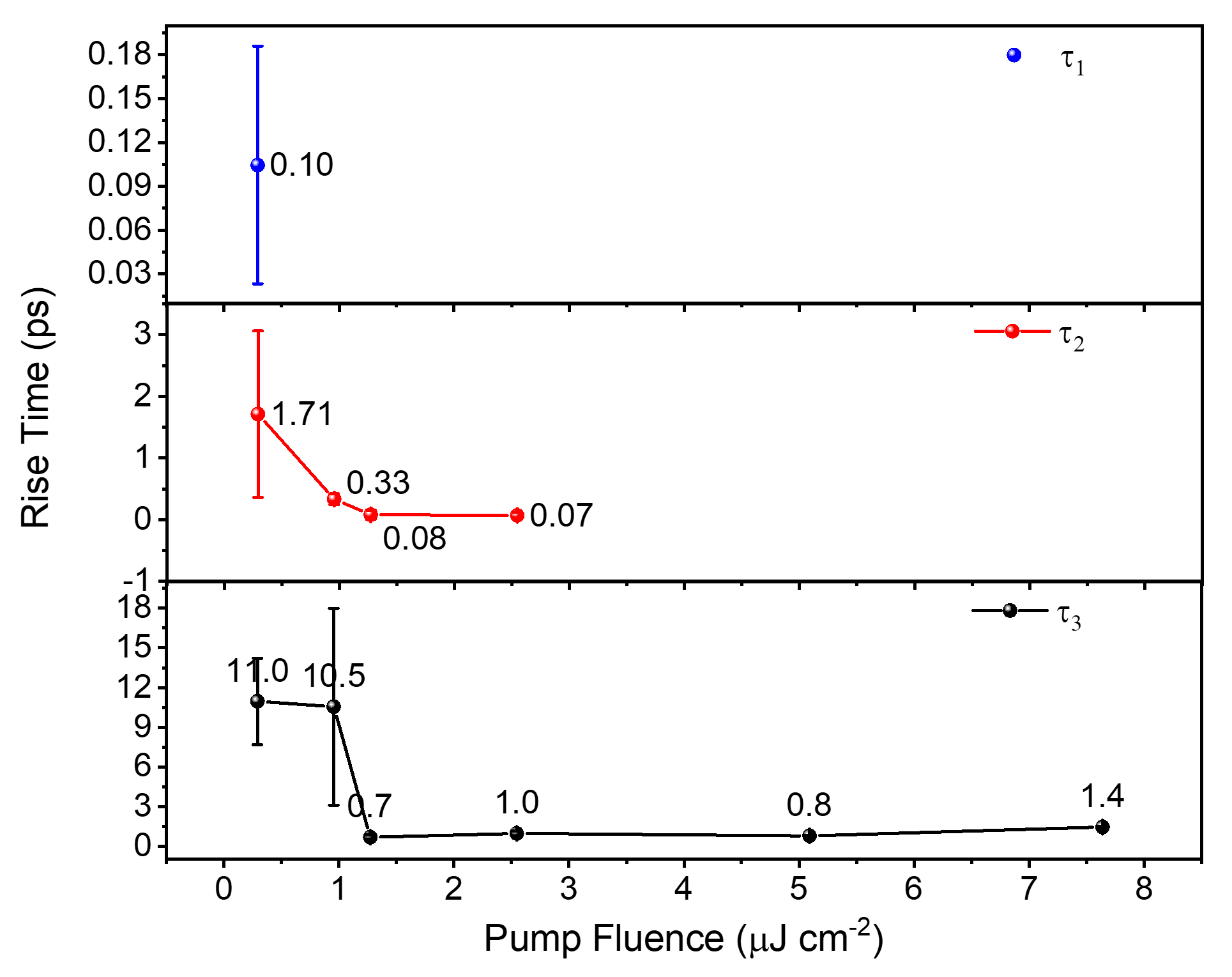
**

**Fig. S14 Rise time versus pump fluence extracted from *n*=∞ phase.** With the increase of pump fluence, population saturation of the states in *n*=∞ phase blocks the exciton localization process and accelerate the rising time, and the rising process persists at high pump fluence.

**Supplementary Note 1. The fitting of temperature dependent broadening PL emission.**

The broadening of temperature dependent PL is in connection with the interference of lattice scattering with electron motion in various ways. The comprehensive broadening PL can be described by the sum of broadening contributions, as following equation:

$$\Gamma\left( T \right)=\Gamma_{0}+\Gamma_{LA}+\Gamma_{LO}+\Gamma_{imp}{=\Gamma_{0}+\gamma}_{LA}T+\frac{\Gamma_{LO}}{\exp\left( \frac{\hbar\bar{\omega}}{k_{B}T} \right)-1}+\Gamma_{imp}exp(-E_{b}/k_{B}T)$$

Where the first term $\Gamma_{0}$ is the temperature-independent inhomogeneous broadening, that is related with the electron-electron interactions, impurities, dislocations and so on. Second term is longitudinal acoustic (LA) phonon scattering induced homogeneous broadening, in which $\gamma_{LA}$ is the coefficient of its LA phonon-exciton (or electron) coupling strength, reflecting the deformation potential interaction that is linearly dependent on temperature. Third term is the broadening contribution of longitudinal optical (LO) phonon-exciton (or electron) coupling describing the Fröhlich interaction between electrons and the longitudinal electric field generated by LO phonon modes, where $\Gamma_{LO}$ is the coupling strength of LO phonon-exciton (or electron), $\hbar\bar{\omega}$ is corresponding phonon energy, relating to the frequency of weakly dispersive LO phonon branch. The last term is the broadening on account of scattering of ionized impurities which is determined by their average binding energy E_b_. According to the previous report, since the perovskite is a polar semiconductor, the contribution of $\Gamma_{LA}$ and $\Gamma_{imp}$ is insignificant comparing with that of $\Gamma_{0}$ and $\Gamma_{LO}$, so these two terms can be neglected here^1-3^. Furthermore, the variation of phonon energy as a function of temperature is also ignored.

**Supplementary Note 2. Temperature dependent band gap energy.**

The band gap energies of various dimensional phases were also extracted and analyzed. As shown in PL spectrum, the band gap of all phases (*n*=3~∞) exhibit a monotonic blue shift from 67 to 297 K, which is consistent with the previous study^4^. Generally, the variation of temperature dependent band gap energy of semiconductors is determined by the combination of thermal expansion and electron-phonon interaction, in which the thermal expansion changes the lattice constant to alter the electronic band structure, while the electron-phonon interaction would cause a change in electronic band structure via lattice vibration. Commonly, the electron-phonon interaction dominated band gap variations exhibit redshift, which is typically modeled by using Varshni empirical formular. So, in the case of our mixed dimensional perovskite, the thermal expansion may dominate the band gap evolution and lead to a linear blue-shift to the band gap.

The temperature dependent band gap energy can be expressed as^5^:

$$\frac{\partial E_{g}}{\partial T}=\frac{\partial E_{g}}{\partial V}\frac{\partial V}{\partial T}+\sum_{j,\vec{q}} \left( \frac{\partial E_{g}}{\partial n_{j,\vec{q}}} \right)\left( n_{j,\vec{q}}+\frac{1}{2} \right)$$

where $n_{j,\vec{q}}$ is the phonon number of the j_th_ branch with the wave number of q, and the phonons normally follows the Bose-Einstein distribution: $n_{j,\vec{q}}=\frac{1}{\exp\left( {\hbar\omega_{j,\vec{q}}}/{k_{B}T} \right)-1}$. In the above equation, the first term is attributed to the thermal expansion of the crystal lattice that resulted from the anharmonicity of interatomic potentials, where the ${\partial V}/{\partial T}$ is deformation potential and ${\partial E_{g}}/{\partial V}$ is the expansion coefficient. The expansion coefficient of a semiconductor can be approximated as a temperature-independent constant depending on the specific structure of band gap. The second term reflect the contribution from the electron-phonon interaction, including all possible phonon modes within the entire Brillouin zone, which makes it difficult to accurately calculate the temperature dependent band gap evaluation due to the complicated electron-phonon interaction. Actually, in the previous studies, only a few dominant phonon modes are taken into consideration, such as the most frequently used one branch of acoustic and optical phonons. As previous report, in the case of the quasi-2D nanoplate (OABr-CsPbBr_3_) and temperature range of 77-297 K, the electron-phonon interaction is dominated by the only one-oscillator model of longitudinal optical phonon scattering via Fröhlich coupling and the acoustic phonon scattering can be negligible^6^. So, we employed the simplified model in our fitting, the equation can be expressed as following:

$$E_{g}\left( T \right)=E_{0}+A_{TE}T+A_{EP}\left[ \frac{2}{exp\left( \Theta/T \right)-1}+1 \right]$$

Where $E_{0}$ is the unrenormalized band gap energy, and the band gap at 0 K is described as $E_{0}+ A_{EP}$ with the consideration of the quantum factor in the Bose-Einstein distribution. $A_{TE}$ and $A_{EP}$ is the interaction strength coefficient of thermal expansion and electron-phonon coupling, respectively. $\Theta$ is the value corresponding to the average phonon energy.

Our experimental data of temperature dependent band gap energy is well fitted by applying the above equation. The obtained result of $A_{EP}$ (-42.41 meV) and $\Theta$ (438 K) of *n*=∞ phase in our multiple dimensional perovskites is comparable with that of CsPbBr_3_ in the previous report^7^. And the relative exciton-phonon coupling strength in the *n*=3 and *n*=∞ phases is consistent with the result calculated from the FWHM versus temperature.

**References**

1. Wright, A. D. et al. Electron-phonon coupling in hybrid lead halide perovskites. *Nat. Commun.* **7**, 11755 (2016).

2. Wei, K. et al. Temperature-dependent excitonic photoluminescence excited by two-photon absorption in perovskite CsPbBr_3_ quantum dots. *Opt. Lett.* **41**, 3821-3824 (2016).

3. Wolf, C., Lee, T.-W. Exciton and lattice dynamics in low-temperature processable CsPbBr_3_ thin-films. *Mater. Today Energy* **7**, 199-207 (2018).

4. Long, H. et al. Exciton-phonon interaction in quasi-two dimensional layered (PEA)_2_(CsPbBr_3_)_n-1_PbBr_4_ perovskite. *Nanoscale* **11**, 21867-21871 (2019).

5. Yu, C. et al. Temperature dependence of the band gap of perovskite semiconductor compound CsSnI_3_. *J. Appl. Phys.* **110**, 063526 (2011).

6. Peng, S. et al. Suppressing strong exciton-phonon coupling in blue perovskite nanoplatelet solids by binary systems. *Angew. Chem. Int. Ed.* **59**, 22156-22162 (2020).

7. Shi, Y. Q. et al. Synthesis and stability of CsPbBr_3_ perovskite nanorods with high optical gain. *Chin. J. Lasers* **47**, 0701024 (2020).
